# Association of Antipsychotic Polypharmacy vs Monotherapy With Psychiatric Rehospitalization Among Adults With Schizophrenia

**DOI:** 10.1001/jamapsychiatry.2018.4320

**Published:** 2019-02-20

**Authors:** Jari Tiihonen, Heidi Taipale, Juha Mehtälä, Pia Vattulainen, Christoph U. Correll, Antti Tanskanen

**Affiliations:** 1 Department of Clinical Neuroscience, Karolinska Institutet, Stockholm, Sweden; 2 Department of Forensic Psychiatry, University of Eastern Finland, Niuvanniemi Hospital, Kuopio, Finland; 3 Center for Psychiatry Research, Stockholm City Council, Stockholm, Sweden; 4 School of Pharmacy, University of Eastern Finland, Kuopio, Finland; 5 EPID Research Oy, Espoo, Finland; 6 Department of Psychiatry and Molecular Medicine, Hofstra Northwell School of Medicine, Hempstead, New York; 7 Department of Psychiatry, The Zucker Hillside Hospital, Glen Oaks, New York; 8 Department of Child and Adolescent Psychiatry, Charité Universitätsmedizin, Berlin, Germany,; 9 National Institute for Health and Welfare, The Impact Assessment Unit, Helsinki, Finland

## Abstract

**Importance:**

The effectiveness of antipsychotic polypharmacy in schizophrenia relapse prevention is controversial, and use of multiple agents is generally believed to impair physical well-being.

**Objective:**

To study the association of specific antipsychotic combinations with psychiatric rehospitalization.

**Design, Setting, and Participants:**

In this nationwide cohort study, the risk of psychiatric rehospitalization was used as a marker for relapse among 62 250 patients with schizophrenia during the use of 29 different antipsychotic monotherapy and polypharmacy types between January 1, 1996, and December 31, 2015, in a comprehensive, nationwide cohort in Finland. We conducted analysis of the data from April 24 to June 15, 2018. Rehospitalization risks were investigated by using within-individual analyses to minimize selection bias.

**Main Outcomes and Measures:**

Hazard ratio (HR) for psychiatric rehospitalization during use of polypharmacy vs during monotherapy within the same individual.

**Results:**

In the total cohort, including 62 250 patients, 31 257 individuals (50.2%) were men, and the median age was 45.6 (interquartile range, 34.6-57.9) years. The clozapine plus aripiprazole combination was associated with the lowest risk of psychiatric rehospitalization in the total cohort, being superior to clozapine, the monotherapy associated with the best outcomes, with a difference of 14% (HR, 0.86; 95% CI, 0.79-0.94) in the analysis including all polypharmacy periods, and 18% in the conservatively defined polypharmacy analysis excluding periods shorter than 90 days (HR, 0.82; 95% CI, 0.75-0.89; *P* < .001). Among patients with their first episode of schizophrenia, these differences between clozapine plus aripiprazole vs clozapine monotherapy were greater (difference, 22%; HR, 0.78; 95% CI, 0.63-0.96 in the analysis including all polypharmacy periods, and difference, 23%; HR, 0.77; 95% CI, 0.63-0.95 in the conservatively defined polypharmacy analysis). At the aggregate level, any antipsychotic polypharmacy was associated with a 7% to 13% lower risk of psychiatric rehospitalization compared with any monotherapy (ranging from HR, 0.87; 95% CI, 0.85-0.88, to HR, 0.93; 95% CI, 0.91-0.95; *P* < .001). Clozapine was the only monotherapy among the 10 best treatments. Results on all-cause and somatic hospitalization, mortality, and other sensitivity analyses were in line with the primary outcomes.

**Conclusions and Relevance:**

Combining aripiprazole with clozapine was associated with the lowest risk of rehospitalization, indicating that certain types of polypharmacy may be feasible in the treatment of schizophrenia. Because add-on treatments are started when monotherapy is no longer sufficient to control for worsening of symptoms, it is likely that the effect sizes for polypharmacy are underestimates. Although the results do not indicate that all types of polypharmacy are beneficial, the current treatment guidelines should modify their categorical recommendations discouraging all antipsychotic polypharmacy in the maintenance treatment of schizophrenia.

## Key Points

**Question:**

Are there specific antipsychotic combinations that are superior to monotherapies in the maintenance treatment of schizophrenia?

**Findings:**

This cohort study on 62 250 individuals with schizophrenia with up to 20-year follow-up used within-individual analysis to minimize selection bias and showed that antipsychotic polypharmacy in general was associated with slightly lower risk of psychiatric rehospitalization than monotherapy. Clozapine plus aripiprazole combination was associated with the best outcome, having 14% to 23% lower risk of rehospitalization than clozapine alone, which was the monotherapy associated with the best outcomes.

**Meaning:**

The findings of this study suggest that certain types of polypharmacy may be associated with fewer rehospitalizations than monotherapies.

## Teaser

This national cohort study examines the use of polypharmacy vs monotherapy as maintenance treatment among adult patients with schizophrenia who have been rehospitalized for psychiatric treatment in Finland.

## Introduction

Antipsychotic polypharmacy is used among up to 30% of patients with schizophrenia.^1^ The use of antipsychotic polypharmacy has raised concern owing to the lack of evidence for its efficacy and safety as well as variable justifications and practice patterns.^2,3,4,5,6,7^ Meta-analyses of randomized clinical trials (RCTs) have shown mixed results,^8,9,10,11,12^ possibly because of shortcomings owing to low number of participants and lacking separations of high- vs low-quality studies. The most-recent meta-analysis without these limitations concluded that data from high-quality studies show beneficial outcomes only for negative symptom reduction with aripiprazole augmentation.^12^ Short-term symptom reduction, used as the primary outcome in RCTs, is an important measure for effectiveness of antipsychotic treatment. However, schizophrenia is a lifelong illness, and long-term outcome, including relapse prevention and avoidance of adverse physical morbidity and mortality effects owing to long-term antipsychotic load, is an even more important issue for the patients’ well-being.^13,14^ Conducting an RCT on these outcomes would require several thousands of patient-years, which is probably the reason why no such studies have been done.

Observational studies can overcome this problem by using large electronic databases. Results from 1 large observational study have shown that any antipsychotic polypharmacy was associated with an approximately 40% lower risk of rehospitalization and death compared with any monotherapy,^15^ but the major problem in observational studies is residual confounding related to selection bias. This limitation can be eliminated by using within-individual analyses in which each patient is used as his or her own control.^16,17,18^ To our knowledge, no observational study has been published on the comparative effectiveness of antipsychotic combinations vs monotherapies using this method. We aimed to study this issue, using within-individual analyses, in a nationwide cohort including all patients with schizophrenia in Finland.

## Methods

### Study Population

This study was based on a cohort of all persons with schizophrenia treated in the inpatient setting during 1972-2014 in Finland, identified from the Hospital Discharge register maintained by the National Institute of Health and Welfare. Data were also retrieved from the National Prescription register (maintained by Social Insurance Institution, 1995-2015) and National Death register (1972-2015). The Hospital Discharge register includes all inpatient hospital stays in Finland, recorded for all residents. The detailed information on the study population is given in the eMethods in the Supplement. The follow-up started January 1, 1996, for the prevalent cohort and at the first discharge from inpatient care for the incident cases. The follow-up time ended at death or December 31, 2015, whichever occurred first. We conducted analysis of the data from April 24 to June 15, 2018.

The research project was approved by the ethics committee of the Finnish National Institute for Health and Welfare. Further permissions were granted by pertinent institutional authorities at the National Institute for Health and Welfare of Finland, the Social Insurance Institution of Finland, and Statistics Finland. The study was registry based and no contact was made with the participants of the study, and therefore according to Finnish legislation informed consents were not needed.

### Exposure

Antipsychotic dispensations were derived from the National Prescription register, defined by Anatomical Therapeutic Chemical classification code N05A, excluding lithium.^19^ The register includes reimbursed drug dispensations for the entire Finnish population but does not include drugs used during hospital stays. Data on dispensed drug and amount, also recorded in defined daily doses (World Health Organization^19^), were used for this study. Drug dispensations were modeled with PRE2DUP modeling, a method used to define drug use periods (ie, when drug use started and ended).^20^ The detailed indication of the drug use modeling is shown in the eMethods in the Supplement.

### Outcomes

Psychiatric rehospitalization (*International Classification of Diseases, Tenth Revision* codes F20-F29 as main diagnoses) and all-cause hospitalization were the primary outcome measures. Sensitivity analyses were conducted among incident (first-episode of schizophrenia) patients and for antipsychotic polypharmacy periods excluding the first 90 days of overlap. Hospitalization owing to physical illness and mortality were included as secondary outcomes to take into account that antipsychotic use may have adverse effects on physical health.

### Covariates

The within-individual study design is based on the comparison of different time periods for the same person. Thus, all time-invariant covariates, such as sex, age, time since illness onset, comorbidities, and number of previous psychiatric hospitalizations at cohort entry, are controlled for in the design, and only time-varying covariates are adjusted for in the statistical analysis. Time-varying covariates were the order of antipsychotic exposures, time since cohort entry, and use of other psychotropic drugs (ie, antidepressants, benzodiazepines, lithium, mood stabilizers, sedatives). In this study, grouping of antipsychotics was not identical with the previous study,^18^ resulting in minimally different hazard ratios (HRs) for monotherapies.

The traditional Cox proportional hazards regression models (between-individual analysis) were adjusted for sex, age at cohort entry, year of cohort entry, time since diagnosis, number of prior psychiatric hospitalizations, comorbidities, and drug use, as described in eTable 1 in the Supplement.

### Statistical Analysis

Hospitalization-based outcomes (psychiatric and all-cause hospitalization) were treated as recurrent events and analyzed with a stratified Cox proportional hazards regression model.^16^ In this within-individual design, each patient formed his or her own stratum, and follow-up time was reset to 0 after each outcome event (eFigure 1 in the Supplement). Persons who had both variation in exposure and experienced an outcome event during the follow-up contributed to within-individual analysis. The main analysis compared use of the following drugs in monotherapy and as 2-drug combinations with time when no antipsychotic was used: oral risperidone, quetiapine, clozapine, olanzapine, aripiprazole, and other oral formulations, as well as any long-acting injectable agent. These analyses were conducted in the prevalent cohort (including all patients) and in the incident cohort, including only patients with first-episode, and for both psychiatric and all-cause hospitalization as an outcome event.

Sensitivity analyses were conducted by censoring the first 90 days from antipsychotic use to retrieve time of conservatively defined polypharmacy (ie, excluding switches between monotherapies). This censoring was also conducted for monotherapy periods in an identical way. Also, traditional multivariate-adjusted Cox proportional hazards regression analyses were conducted for all outcome events, and these models were adjusted for covariates provided in eTable 1 in the Supplement. The level of statistical significance was set at *P* < .0017 according to Bonferroni correction (0.05/29 = .0017).

We used the traditional multivariate-adjusted Cox proportional hazards regression as secondary between-individual analyses. In within-individual analysis, only individuals with variation in the exposure (monotherapy, polypharmacy, no antipsychotic) and outcome (rehospitalization) contributed to the model, whereas, for between-individual analysis, all patients contributed to the model. The analyses were conducted using R, version 3.1.1 (R Foundation).

## Results

In the total cohort, including 62 250 patients, 31 257 individuals (50.2%) were men, and the median age was 45.6 (interquartile range, 34.6-57.9) years. The baseline characteristics of the cohort are reported in the Table. The follow-up time in this study was 20 years or less, with a median time of 14.1 years (interquartile range [IQR], 6.9-20.0 years) in the prevalent cohort and 10.1 years (IQR, 5.0-14.3) in the incident cohort (Table). During the follow-up, 58.8% (n = 36 631) of the prevalent cohort and 57.9% (n = 5045) of the incident cohort were readmitted for psychiatric inpatient care. Concerning rehospitalization rates after discontinuation of medication, the median time was 211 (IQR, 68-566) days.

**Table.** Characteristics of the Prevalent and Incident Cohorts and Hospitalizations During Follow-up

Characteristic	Cohort, No. (%)	
	Prevalent (n = 62 250)	Incident (n = 8719)
Age at baseline, y		
≤24	5368 (8.6)	1844 (21.2)
25-34	10 748 (17.3)	2297 (26.3)
35-44	13 996 (22.5)	1417(16.3)
45-54	13 767 (22.1)	1266 (14.5)
55-64	8833 (14.2)	763 (8.8)
≥65	9538 (15.3)	1132 (13.0)
Median age (IQR), y	45.6 (34.6-57.9)	36.2 (26.2-52.3)
Men	31 257 (50.2)	4898 (56.2)
No. of all-cause hospitalizations		
0	8617 (13.8)	1748 (20.0)
1	7948 (12.8)	1443 (16.6)
2-4	17 194 (27.6)	2603 (29.9)
5-8	12 423 (20.0)	1520 (17.4)
≥9	16 068 (25.8)	1405 (16.1)
No. of all-cause hospitalizations per person, median (IQR)	4 (1-9)	3 (1-6)
No. of psychiatric hospitalizations		
0	25 619 (41.2)	3674 (42.1)
1	10 233 (16.4)	1615 (18.5)
2-4	13 490 (21.7)	1980 (22.7)
5-8	6273 (10.1)	805 (9.2)
≥9	6635 (10.7)	645 (7.4)
No. of psychiatric hospitalizations per person, median (IQR)	1 (0-4)	1 (0-3)
Follow-up time, median (IQR), y	14.1 (6.9-20.0)	10.1 (5.0-14.3)

Abbreviation: IQR, interquartile range.

Corresponding values for all-cause hospitalization were 86.2% (n = 53 633) of the prevalent cohort and 80.0% (n = 6971) of the incident cohort. A total of 67.2% (n = 41 812) of the patients in the total cohort and 54.1% (n = 4717) of those in the incident cohort used antipsychotic polypharmacy during the follow-up, and 57.5% (n = 35 793) of the prevalent cohort and 41.6% (n = 3627) of the incident cohort were exposed to antipsychotic polypharmacy for at least 90 days. Median doses of specific antipsychotics used in the prevalent and incident cohorts are described in eTable 2 in the Supplement.

**Figure 1.**
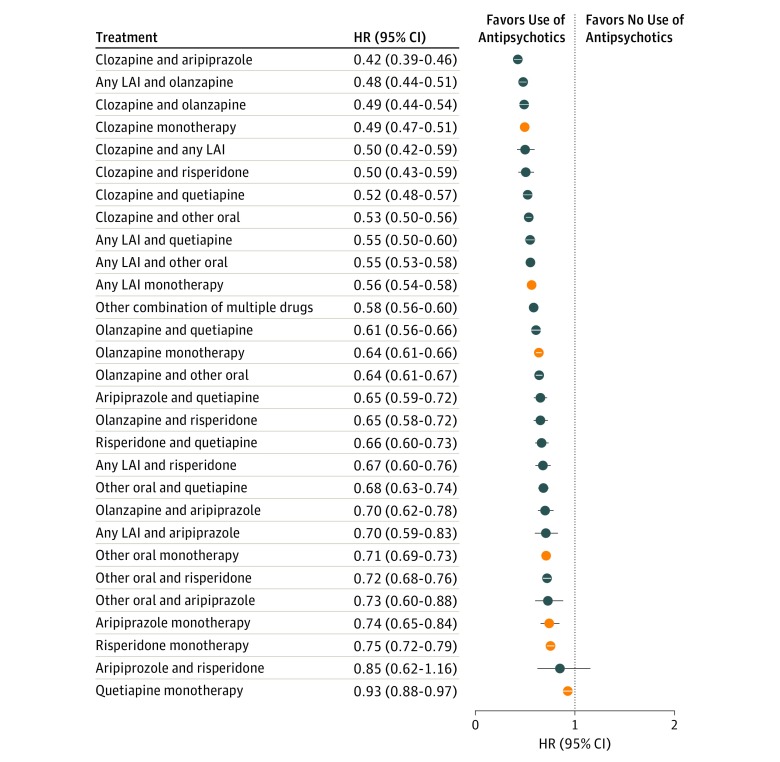
Risk of Psychiatric Rehospitalization During Specific Treatments Compared With No Antipsychotic Use in the Prevalent Cohort (Within-Individual Analysis) HR indicates hazard ratio; LAI, long-acting injectable agent. Orange markers indicate monotherapies.

**Figure 2.**
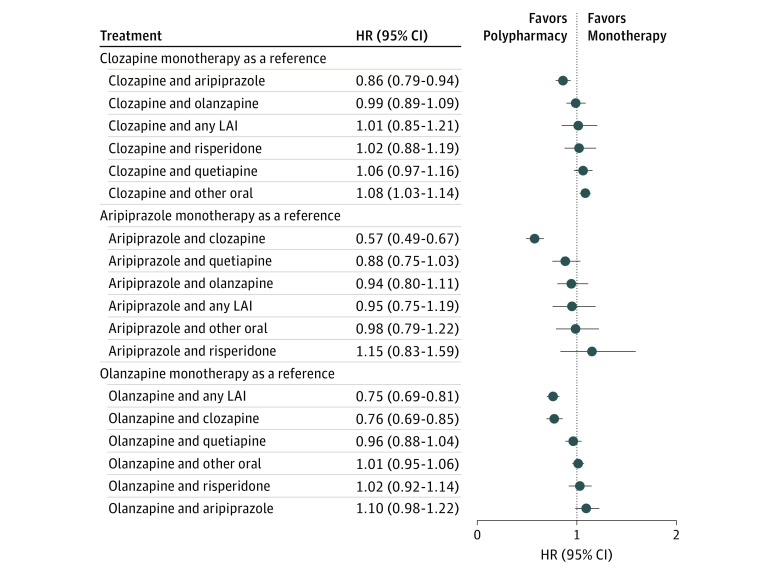
Risk of Psychiatric Rehospitalization in the Total Cohort, Compared With Clozapine, Aripiprazole, and Olanzapine Monotherapy (Within-Individual Analysis) HR indicates hazard ratio; LAI, long-acting injectable agent.

**Figure 3.**
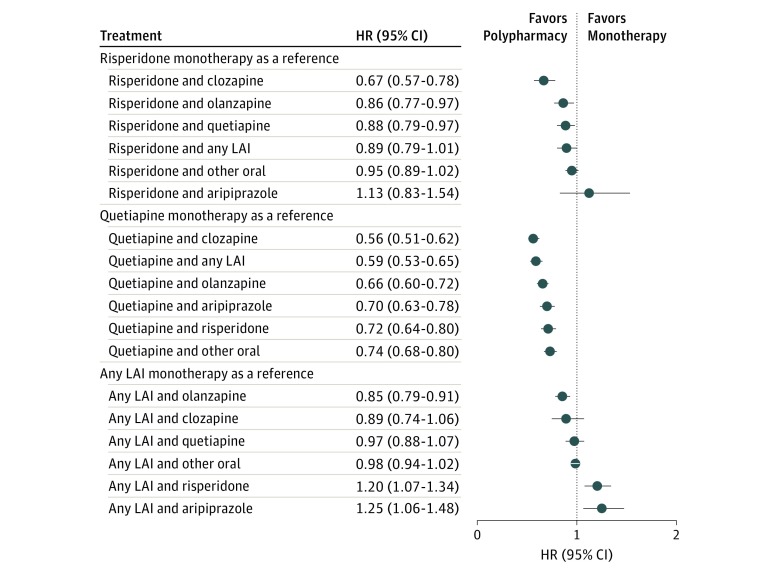
Risk of Psychiatric Rehospitalization in the Total Cohort, Compared With Risperidone, Quetiapine, and Any Long-Acting Injectable Agent (LAI) Monotherapy (Within-Individual Analysis) HR indicates hazard ratio; LAI, long-acting injectable agent.

The overall view on the risks of psychiatric rehospitalization for specific treatments in the total cohort are presented in Figure 1 and eTable 3 in the Supplement. The corresponding results using monotherapies as the reference are shown in Figure 2 and Figure 3. The lowest risk of rehospitalization was observed for clozapine plus aripiprazole polypharmacy (Figure 2), being 14% lower (HR, 0.86; 95% CI, 0.79-0.94; *P* < .001) than that for clozapine, the monotherapy associated with the best outcomes. Among individuals who used clozapine both as monotherapy and polypharmacy, the mean clozapine dose was 426 mg/d during monotherapy and 399 mg/d during polypharmacy. When other specific polypharmacy combinations, excluding the composite group of any long-acting injectable agents, were compared with the better antipsychotic component of each combination, no combination was statistically superior to monotherapy when strict Bonferroni correction was applied. eTable 4 in the Supplement reports the outcome noted when any other antipsychotic was added to aripiprazole, clozapine, olanzapine, quetiapine, and risperidone.

The risk of psychiatric hospitalization, all-cause hospitalization, or death was not decreased significantly by adding any miscellaneous antipsychotic (most of those other than aripiprazole) to clozapine. However, including any other agent with quetiapine, which had the worst monotherapy response, resulted in a better outcome. At an aggregate level, the risk of psychiatric rehospitalization was 7% lower during any polypharmacy than any monotherapy period without censoring the first 90-day periods of antipsychotic treatment (HR, 0.93; 95% CI, 0.91-0.95; *P* < .001). The HR for all-cause hospitalization was 0.91 (95% CI, 0.89-0.92; *P* < .001), and for death, 0.76 (95% CI, 0.73-0.79; *P* < .001).

eFigure 2 and eTable 5 in the Supplement provide the results for psychiatric rehospitalization when the first 90 days were censored from all treatment periods to eliminate potential artificial polypharmacy periods that may have occurred when one antipsychotic was switched to another. The superiority of clozapine plus aripiprazole (which had HR, 0.55; 95% CI, 0.51-0.61 vs no antipsychotic) over clozapine monotherapy (which had HR, 0.68; 95% CI, 0.66-0.70 vs no antipsychotic) was even greater (difference, 18%; HR, 0.82; 95% CI, 0.75-0.89; *P* < .001) in this conservatively defined polypharmacy analysis. In this analysis, the risk of rehospitalization was 13% lower during any polypharmacy than any monotherapy treatment (HR, 0.87; 95% CI, 0.85-0.88; *P* < .001).

**Figure 4.**
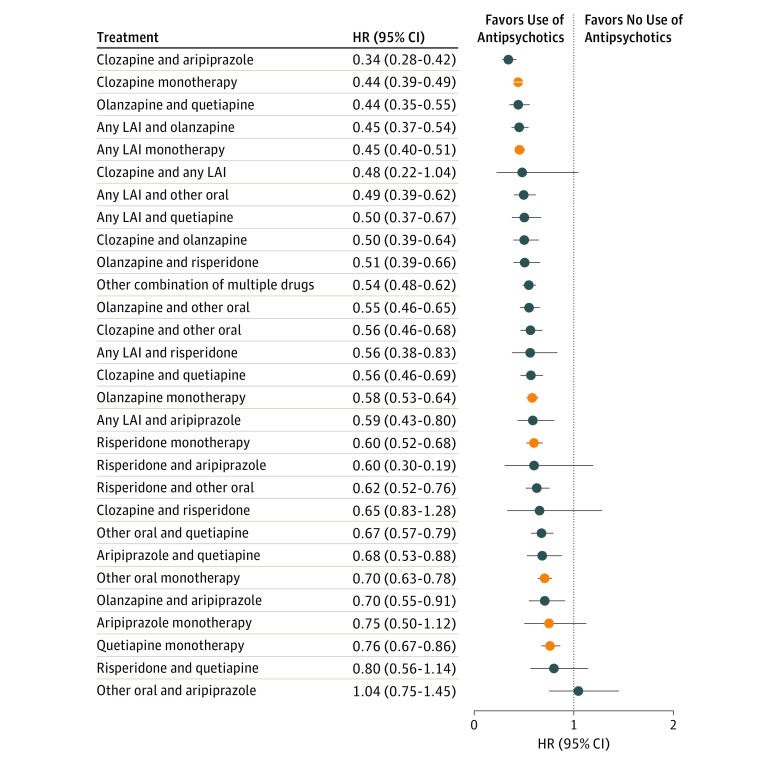
Risk of Psychiatric Hospitalization During Specific Treatments Compared With No Antipsychotic Use in the Incident Cohort (Within-Individual Analysis) HR indicates hazard ratio. LAI, long-acting injectable agent. Orange markers indicate monotherapies.

The risk of all-cause hospitalization is shown in eFigure 3 and eTable 6 in the Supplement. Again, clozapine plus aripiprazole was associated with a substantially better outcome than any other treatment. Figure 4 and eTable 7 in the Supplement show the results for the first-episode group with no antipsychotic use as reference. The superiority of the clozapine plus aripiprazole combination over any other treatments was even more robust than in the total cohort. When clozapine, the monotherapy associated with the best outcomes, was used as reference, the HR for clozapine plus aripiprazole combination was 0.78 (95% CI, 0.63-0.96) in the analysis including all polypharmacy periods, and 0.77 (95% CI, 0.63-0.95) in conservatively defined polypharmacy analysis. The risk of hospitalization owing to physical illness is given in eFigure 4 and eTable 8 in the Supplement, showing the lowest risks for polypharmacy and long-acting injectable agent monotherapy.

The results for all-cause mortality are presented in eFigure 5 and eTable 9 in the Supplement. Among 29 different treatments, all monotherapies except clozapine were among the 10 worst, although all treatments were associated with a 50% or more lower risk of death compared with no antipsychotic use. The results for psychiatric hospitalizations with between-individual analysis including all patients, as well as also those without polypharmacy or hospitalizations, are presented in eFigure 6, eFigure 7, eTable 10, and eTable 11 in the Supplement. Clozapine and clozapine plus aripiprazole were again among the most favorable treatments.

## Discussion

To our knowledge, this is the first study on the long-term use of antipsychotic polypharmacy in schizophrenia. It is nearly impossible to conduct an RCT including tens of thousands of patient-years to achieve sufficient statistical power. Therefore, observational studies are the only way to investigate long-term comparative outcomes. The major shortcoming in observational studies is selection bias, because treatments are not chosen on a random basis. In this study, we used within-individual analyses in which each patient is used as his or her own control to minimize selection bias. We observed that when treatment for the same patient was switched back and forth between monotherapy and polypharmacy, the use of aripiprazole plus clozapine was associated with a 14% to 23% lower risk of psychiatric or all-cause hospitalization compared with clozapine monotherapy. Clozapine was the monotherapy associated with the best outcomes, and clozapine plus aripiprazole was associated with significantly better outcome than any other antipsychotic treatment, either as monotherapy or polypharmacy. Quetiapine was the least successful monotherapy, as has been observed also in previous Swedish and Finnish studies.^17,18^

One possible explanation for the superiority of polypharmacy is that, in the real-world setting, treatment adherence is poor,^21,22,23^ and, if the patient has prescriptions for 2 antipsychotics, he or she may use at least 1 of them. However, other data suggest that the more medications or doses used, the more difficult it is for patients to adhere to the treatment regimen.^22^ Regarding the clozapine plus aripiprazole combination, the clozapine dose was only slightly lower during polypharmacy than during monotherapy, which suggests that reduction of the dose was not the major explanation for the better outcome. However, it is plausible that the different types of receptor profiles result in beneficial effects. For example, in a meta-analysis, addition of the partial dopamine D_2_ receptor agonist aripiprazole to clozapine therapy improved negative symptoms and reduced several adverse effects, such as weight gain and increased prolactin level, whereas a combination of 2 dopamine D_2_ antagonists was associated with greater prolactin elevation but less insomnia.^12^ Such a reduced adverse effect burden could also increase adherence.

The mean daily doses of risperidone and quetiapine were low in our cohort, showing what happens in a total, nationwide cohort of patients with schizophrenia. It is probable that the prescribed doses had been higher, but the observed consumed doses (calculated on the basis of successively filled prescriptions in pharmacies) indicated the doses that patients actually used, and it is plausible that they titrated the doses to the level that they could tolerate. Therefore, patients may be willing to use 2 antipsychotics in relatively low doses but refuse to use high or moderate doses of monotherapy even though the total defined daily dose would be lower during monotherapy. Therefore, it would be misleading to presume that effectiveness (efficacy plus tolerability) would correlate with the total antipsychotic dose expressed as defined daily doses or chlorpromazine equivalents.

Our results revealed that, in general, antipsychotic polypharmacy was associated with an approximately 10% lower relative risk of psychiatric rehospitalization (corresponding to an approximately 6% lower absolute risk with an approximately 60% rehospitalization rate in the cohort) compared with antipsychotic monotherapy, translating into a number needed to treat of 10 to 20, which is generally considered clinically meaningful. This effect size is larger than the effect size of statin treatments for the prevention of cardiovascular incidents.^24^ To reach statistical significance for a result of this magnitude, the minimum number of patients to compare 2 groups of treatments is approximately 1000. This sample size requirement for a long-term study is probably a major reason why RCTs are difficult to conduct to answer questions about the relative effectiveness of several active treatments in the maintenance management of schizophrenia.

In addition, secondary analyses on hospitalization owing to physical illness and mortality showed better outcomes for antipsychotic combinations than for monotherapy. Because add-on treatments are started when monotherapy is no longer sufficient to control for worsening of symptoms, it is likely that the effect sizes for the superiority of antipsychotic polypharmacy over monotherapy are underestimates. The results obtained with 90-day omission in each treatment period (conservatively defined polypharmacy analysis excluding switch periods) were more robust than those without 90-day omission and are probably more accurate estimates on the results of polypharmacy. The results from between-individual analyses, including all patients, showed similar rank order as within-individual analyses. This finding indicates that the clozapine plus aripiprazole combination is associated with the best outcome in the entire population of Finnish patients with schizophrenia. The results may generalize to other high-income countries with a majority white population but not necessarily to other societies. Only 1 other study has compared multiple antipsychotic combinations in relapse prevention.^15^ Our results are in line with those of Katona et al,^15^ showing a lower risk of rehospitalization during antipsychotic polypharmacy compared with monotherapy. However, our effect sizes were smaller, which may be explained by use of within-individual analysis, which minimizes selection bias.

Our results on mortality are in line with those of previous cohort studies, showing lower mortality during antipsychotic polypharmacy than monotherapy.^15,25,26^ Two small studies have reported a positive correlation between antipsychotic polypharmacy and mortality: one investigated the maximum number of concomitant antipsychotics used during a 10-year follow-up (n = 88)^27^ and the other studied the number of antipsychotics used (n = 99) at baseline of a 17-year follow-up.^28^ Because these studies were based on case records, their results may reveal associations between mortality and the number of antipsychotic prescriptions given to patients several years before death rather than actual past or current use based on filling of prescriptions. Our outcomes did not include functioning or quality of life, but these outcomes are reflected to some extent in mortality and psychiatric and somatic hospitalization rates.

### Strengths and Limitations

This study has both strengths and limitations. We included all hospital-treated patients in Finland with up to 20-year follow-up and used within-individual analysis to minimize selection bias. In addition, time-varying covariates, such as time since cohort entry and order of exposures (ie, switch from monotherapy to polypharmacy vs switch from polypharmacy to monotherapy), were adjusted in the analysis. One limitation is that our main outcomes were risk of rehospitalization owing to psychiatric or somatic reasons, and our database did not include information on symptoms, reason for polypharmacy, quality of life, and level of functioning. We did not have information on concomitant psychosocial treatments, but it is unlikely that there would be any systematic differences between polypharmacy and monotherapy in this regard. In addition, clozapine and long-acting injectable agent treatments require regular contact with health care staff, which may be associated with better outcomes, but it is unlikely that the increased contact would explain the difference between polypharmacy and monotherapy. Another limitation of the study was that the results may generalize to only high-income countries with a majority white population. An additional limitation was protopathic-type bias (ie, the fact that add-on treatments are started when symptoms become worse). Therefore, the effect sizes for polypharmacy are probably underestimated.

## Conclusions

Our results suggest that patients had the lowest risk of psychiatric or all-cause hospitalization when they received combination therapy with clozapine plus aripiprazole, which was significantly superior to clozapine, which was the monotherapy associated with the best outcomes. These results indicate that rational antipsychotic polypharmacy seems to be feasible by using 2 particular antipsychotics with different types of receptor profiles.

Current treatment guidelines state that antipsychotic monotherapy should be preferred and polypharmacy should be avoided if possible. These recommendations reflect the recent evidence in high-quality studies on the acute-phase treatment. However, results from our study suggest that antipsychotic polypharmacy may be superior to monotherapy for maintenance treatment, which has not been examined with RCTs. Therefore, it should be acknowledged that statements about a preferential use of antipsychotic monotherapy for maintenance treatment of schizophrenia lack evidence, and that currently available evidence—although gathered with few nonrandomized cohort studies that have their own limitations—indicates the opposite. Therefore, the current treatment guidelines should modify their categorical recommendations discouraging all antipsychotic polypharmacy in the maintenance treatment of schizophrenia.

## Notes

Supplement. **eMethods.** Population and Exposure **eTable 1.** Covariate Definitions **eTable 2.** Median Doses of Antipsychotics in Defined Daily Doses (DDDs) and Milligrams (mg), With Interquartile Ranges (IQRs) **eTable 3.** Risk of Psychiatric Hospitalization During Specific Treatments Compared With No Antipsychotic Use in the Prevalent Cohort (Within-Individual Analysis) **eTable 4.** The Effect Of Adding Any Other Antipsychotic on Top of Aripiprazole, Clozapine, Olanzapine, Quetiapine, and Risperidone Monotherapy **eTable 5.** Risk of Psychiatric Hospitalization During Specific Treatments Compared With No Antipsychotic Use in the Prevalent Cohort (Within-Individual Analysis Conservatively Defined Polypharmacy) **eTable 6.** Risk of All-Cause Hospitalization During Specific Treatments Compared With No Antipsychotic Use in the Prevalent Cohort (Within-Individual Analysis) **eTable 7.** Risk of Psychiatric Hospitalization During Specific Treatments Compared With No Antipsychotic Use in the Incident Cohort (Within-Individual Analysis) **eTable 8.** Risk of Somatic Hospitalization During Specific Treatments Compared With No Antipsychotic Use (Within-Individual Analysis) **eTable 9.** Risk Of Death During Specific Antipsychotic Treatments Compared With No Use of Antipsychotic in the Total Cohort (Between-Individual Analysis) **eTable 10.** Risk of Psychiatric Hospitalization During Specific Antipsychotic Treatments Compared With No Use of Antipsychotic in the Total Cohort (Between-Individual Analysis) **eTable 11.** Risk of Psychiatric Re-Hospitalization During Specific Treatments Compared With No Antipsychotic Treatment in the Incident Cohort (Between-Individual Analyses) **eFigure 1.** Restructuring of Data for Within Individual Analyses **eFigure 2.** Risk of Psychiatric Hospitalization During Specific Treatments Compared With No Antipsychotic Use in the Prevalent Cohort (Within-Individual Analysis) **eFigure 3.** Risk of All-Cause Hospitalization During Specific Treatments Compared With No Antipsychotic Use in the Prevalent Cohort (Within-Individual Analysis) **eFigure 4.** Risk of Somatic Hospitalization During Specific Treatments Compared With No Antipsychotic Use (Within-Individual Analysis) **eFigure 5.** Risk of Death During Specific Antipsychotic Treatments Compared With No Use of Antipsychotic in the Total Cohort (Between-Individual Analysis) **eFigure 6.** Risk of Psychiatric Hospitalization During Specific Antipsychotic Treatments Compared With No Use of Antipsychotic in the Total Cohort (Between-Individual Analysis) **eFigure 7.** Risk of Psychiatric Re-Hospitalization During Specific Treatments Compared With No Antipsychotic Treatment in the Incident Cohort (Between-Individual Analyses)
