# Supplementary material for: Association of Antipsychotic Polypharmacy vs Monotherapy With Psychiatric Rehospitalization Among Adults With Schizophrenia
Source: JAMA Psychiatry. 2019 Feb 20;76(5):499–507. doi: 10.1001/jamapsychiatry.2018.4320 (PMC6495354; doi:10.1001/jamapsychiatry.2018.4320)
Supplement: Supplement. — eMethods. Population and Exposure eTable 1. Covariate Definitions eTable 2. Median Doses of Antipsychotics in Defined Daily Doses (DDDs) and Milligrams (mg), With Interquartile Ranges (IQRs) eTable 3. Risk of Psychiatric Hospitalization During Specific Treatments Compared With No Antipsychotic Use in the Prevalent Cohort (Within-Individual Analysis) eTable 4. The Effect Of Adding Any Other Antipsychotic on Top of Aripiprazole, Clozapine, Olanzapine, Quetiapine, and Risperidone Monotherapy eTable 5. Risk of Psychiatric Hospitalization During Specific Treatments Compared With No Antipsychotic Use in the Prevalent Cohort (Within-Individual Analysis Conservatively Defined Polypharmacy) eTable 6. Risk of All-Cause Hospitalization During Specific Treatments Compared With No Antipsychotic Use in the Prevalent Cohort (Within-Individual Analysis) eTable 7. Risk of Psychiatric Hospitalization During Specific Treatments Compared With No Antipsychotic Use in the Incident Cohort (Within-Individual Analysis) eTable 8. Risk of Somatic Hospitalization During Specific Treatments Compared With No Antipsychotic Use (Within-Individual Analysis) eTable 9. Risk Of Death During Specific Antipsychotic Treatments Compared With No Use of Antipsychotic in the Total Cohort (Between-Individual Analysis) eTable 10. Risk of Psychiatric Hospitalization During Specific Antipsychotic Treatments Compared With No Use of Antipsychotic in the Total Cohort (Between-Individual Analysis) eTable 11. Risk of Psychiatric Re-Hospitalization During Specific Treatments Compared With No Antipsychotic Treatment in the Incident Cohort (Between-Individual Analyses) eFigure 1. Restructuring of Data for Within Individual Analyses eFigure 2. Risk of Psychiatric Hospitalization During Specific Treatments Compared With No Antipsychotic Use in the Prevalent Cohort (Within-Individual Analysis) eFigure 3. Risk of All-Cause Hospitalization During Specific Treatments Compared With No Antipsychotic Use in the Prevalent Cohort [file jamapsychiatry-76-499-s001.pdf]

Supplementary Online Content

Tiihonen J, Taipale H, Mehtälä J, Vattulainen P, Correll CU, Tanskanen A. Association of antipsychotic polypharmacy vs monotherapy with psychiatric rehospitalization among adults with schizophrenia. *JAMA Psychiatry*. Published online February 20, 2019. doi:10.1001/jamapsychiatry.2018.4320

eMethods. Population and Exposure

eTable 1. Covariate Definitions

eTable 2. Median Doses of Antipsychotics in Defined Daily Doses (DDDs) and Milligrams (mg), With Interquartile Ranges (IQRs)

eTable 3. Risk of Psychiatric Hospitalization During Specific Treatments Compared With No Antipsychotic Use in the Prevalent Cohort (Within-Individual Analysis)

eTable 4. The Effect Of Adding Any Other Antipsychotic on Top of Aripiprazole, Clozapine, Olanzapine, Quetiapine, and Risperidone Monotherapy

eTable 5. Risk of Psychiatric Hospitalization During Specific Treatments Compared With No Antipsychotic Use in the Prevalent Cohort (Within-Individual Analysis Conservatively Defined Polypharmacy)

eTable 6. Risk of All-Cause Hospitalization During Specific Treatments Compared With No Antipsychotic Use in the Prevalent Cohort (Within-Individual Analysis)

eTable 7. Risk of Psychiatric Hospitalization During Specific Treatments Compared With No Antipsychotic Use in the Incident Cohort (Within-Individual Analysis)

eTable 8. Risk of Somatic Hospitalization During Specific Treatments Compared With No Antipsychotic Use (Within-Individual Analysis)

eTable 9. Risk Of Death During Specific Antipsychotic Treatments Compared With No Use of Antipsychotic in the Total Cohort (Between-Individual Analysis)

eTable 10. Risk of Psychiatric Hospitalization During Specific Antipsychotic Treatments Compared With No Use of Antipsychotic in the Total Cohort (Between-Individual Analysis)

eTable 11. Risk of Psychiatric Re-Hospitalization During Specific Treatments Compared With No Antipsychotic Treatment in the Incident Cohort (Between-Individual Analyses)

eFigure 1. Restructuring of Data for Within Individual Analyses

eFigure 2. Risk of Psychiatric Hospitalization During Specific Treatments Compared With No Antipsychotic Use in the Prevalent Cohort (Within-Individual Analysis)

eFigure 3. Risk of All-Cause Hospitalization During Specific Treatments Compared With No Antipsychotic Use in the Prevalent Cohort (Within-Individual Analysis)

eFigure 4. Risk of Somatic Hospitalization During Specific Treatments Compared With No Antipsychotic Use (Within-Individual Analysis)

eFigure 5. Risk of Death During Specific Antipsychotic Treatments Compared With No Use of Antipsychotic in the Total Cohort (Between-Individual Analysis)

eFigure 6. Risk of Psychiatric Hospitalization During Specific Antipsychotic Treatments Compared With No Use of Antipsychotic in the Total Cohort (Between-Individual Analysis)

eFigure 7. Risk of Psychiatric Re-Hospitalization During Specific Treatments Compared With No Antipsychotic Treatment in the Incident Cohort (Between-Individual Analyses)

This supplementary material has been provided by the authors to give readers additional information about their work.

© 2019 American Medical Association. All rights reserved.

eMethods. Population and Exposure

Study population

In Finland, all individuals have a unique personal identity code which was used in the data linkage between databases. Schizophrenia was defined by discharge diagnosis (ICD-10 codes F20, F25; and ICD-9 and ICD-8 codes 295*). Altogether 81,043 persons were hospitalized due to schizophrenia during the study period. After exclusions for diagnoses of dementia (N=1,166 before or at the same time of a schizophrenia diagnosis), those who died during the first hospitalization (N=2,599) and those who died before January 1, 1996 (the time when the incident cohort started and when the follow-up began for the prevalent cohort) (N=15,028), the final prevalent cohort included 62,250 persons with schizophrenia. The incident cohort included persons hospitalized for the first time due to schizophrenia during 1996-2014 (N=23,499) but who had not used antipsychotic drugs during at least one year preceding the index hospitalization, resulting in 8,719 incident cases. The characteristics of this cohort have been reported in previous studies.[1,2]

Exposure

Definition of drug use periods in the PRE2DUP method is based on sliding averages of daily dose and regularity of dispensations; hospitalizations and possible stockpiling of drugs are taken into account. For this study, the modelling was conducted based on drug formulation (according to package information) to separate antipsychotic substances as oral and long-acting injections (LAI). Overlapping use periods were combined to derive time when “any LAI” was used. Oral risperidone, quetiapine, clozapine, olanzapine and aripiprazole were considered separately, and all other orals were pooled together. Since two antipsychotic treatments overlapping are often related to switch and not to actual concomitant use, we also conducted a sensitivity analysis omitting the first 90 days in each polypharmacy and monotherapy treatment period (“conservatively defined polypharmacy” analysis).

© 2019 American Medical Association. All rights reserved. 2

eTable 1. Covariate Definitions.

Covariate: definition	Models utilizing the variable
**Covariates in within–individual models**	
**Order of treatments**: continuously updated in the models, categorized as no treatment, 1st, 2nd, 3rd, >3rd	WI, TC
**Time since cohort entry**: continuously updated in the models, categorized as 0–1, 1–3, >3 years	WI
**Exposure to other psychotropic medications:** Continuously updated variables, each having status "never" until the first purchase of a specific drug occurred. After the first purchase, exposure was "current use" if prior purchases determined that exposure was still on–going and "past use" otherwise (prior purchases too far away in the past). Corresponding ATC codes:<br>- Antidepressants: N06A<br>- Benzodiazepines and related drugs: N05BA 01–22, N05CD, N05CF, N03AE01<br>- Lithium N05AN01<br>- Mood stabilizers: N03AG01, N03AF01, N03AF02, N03AX09, N03AX12, N03AX11<br>- Sedatives: N05C	WI, TC
**Covariates included only in between–individual models**	
**Age**: at cohort entry, categorized as 16–24, 25–34, 35–44, 45–54, 55–64, ≥65	TC
Gender	TC
**Year of cohort entry**: categorized as: 1999 or earlier, 2000–2004, 2005–2009, 2010 or later.	TC
**Time since diagnosis at cohort entry**: categorized as 0–1, 1–3, >3 years	TC
**Number of previous psychiatric inpatient visits**: continuously updated in the models, categorized as 0, 1, 2–4, 5–8, >8	TC
**Non–adherence**: defined at the cohort entry;<br>For prevalent patients (excluding the incident cases): no on–going antipsychotic treatment within 4 months prior to the first inpatient hospitalization during the study period. For incident patients: no filled antipsychotic prescription within one month after the first schizophrenia (ICD–10 codes: F20 or F25) inpatient hospitalization during the study period.	TC
**Exposure to other medications**: Continuously updated variables, each having status "never" until the first purchase of a specific drug occurred. After the first purchase, exposure was "current use" if prior purchases determined that exposure was still on–going and "past use" otherwise (prior purchases too far away in the past). Corresponding ATC codes:<br>- Analgesics: N02A, N02B, N02C, M01A<br>- Anti-parkinson drugs/ anticholinergics: N04AA, N04AB, N04AC<br>- Lipid-modifying agents: C10AA, AB, AC, AD, AX, C10BA01, C10BA02, C10BA03, C10BA06, C1BX01–05<br>- Antidiabetic drugs: A10A, A10BA, BB, BC, BD, BF, BG, BH, BX, A10XA<br>- Drugs used for addictive disorders: N07BB01, N07BB02, N07BB03, N07BB04, N07BB05, N07BC01, N07BC02, N07BC0, N07BC04, N07BC51<br>- Substance abuse: a continuously updated variable with status "no" until the first substance abuse indication occurred and "yes" thereafter. Corresponding ICD–10/ ATC codes: alcohol (F10.1 / N07BB01 – 05), cannabis (F12.1), psychoactive drugs (F19.1), antacids (F55), cocaine (F14.1), opioids (F11.1/ N07BC01 – 04, N07BC51), anxiolytics (F13.1), hallusinogenes (F16.1), solvents (F18.1).	TC

© 2019 American Medical Association. All rights reserved.

**Comorbidities**: continuously updated variables for each of the individual comorbidities presented below, categorized as “ever” vs. “never”.<br>- Cardiovascular disease: I00-I99<br>- Liver disease: K70-K77<br>- Diabetes: E10-E14<br>- Renal disease: N10-N19	TC
**Prior suicide attempt**: continuously updated variable, defined as X60–X84, Z91.5	TC
**Prior use of LAI**: continuously updated variable, “yes” vs. “no”	TC
WI= within–individual Cox model, TC= traditional Cox model, LAI = long–acting injection. Comorbidities according to ICD–10 codes (International Classification of Diseases), medications according to ATC (Anatomical Therapeutic Chemical classification) codes.	

© 2019 American Medical Association. All rights reserved.

eTable 2. Median Doses of Antipsychotics in Defined Daily Doses (DDDs) and Milligrams (mg), With Interquartile Ranges (IQRs). (All exposure periods included).

	**Prevalent cohort**			**Incident cohort**		
	Median dose in DDDs	Median dose in milligrams	IQR for median dose (mg)	Median dose in DDDs	Median dose in milligrams	IQR for median dose (mg)
**Oral antipsychotics**						
aripiprazole	1.00	15.0	14.3–23.0	1.00	15.0	14.0–22.5
clozapine	1.13	337.9	234.8–471.1	1.00	300.9	204.0–418.6
olanzapine	1.40	14.0	9.1–20.0	1.30	13.0	8.7–19.7
quetiapine	0.64	254.5	96.1–508.5	0.55	218.3	75.7–473.2
risperidone	0.56	2.8	1.7–4.3	0.50	2.5	1.6–4.0
**Long–acting injections (LAIs)**						
aripiprazole LAI	1.06	14.1	13.0–14.3	1.06	14.1	13.1–14.3
flupentixol LAI	0.76	3.1	1.5–5.6	0.60	2.4	1.3–5.2
fluphenazine LAI	1.52	1.5	1.1–2.9	1.63	1.63	1.2–3.2
haloperidol LAI	1.29	4.3	3.1–7.1	1.00	3.4	2.1–4.8
olanzapine LAI	1.76	17.6	13.8–21.1	1.80	18.0	13.7–21.2
paliperidone LAI	1.33	3.3	2.6–4.4	1.29	3.2	2.5–4.0
perphenazine LAI	1.01	7.1	4.9–8.9	1.00	7.1	4.5–9.8
pipotiazine LAI	1.19	6.0	3.7–7.1			
risperidone LAI	1.00	2.7	2.1–3.5	0.98	2.6	1.9–3.4
zuclopenthixol LAI	1.02	15.3	12.4–27.2	0.96	14.4	12.1–19.9

© 2019 American Medical Association. All rights reserved.

eTable 3. Risk of Psychiatric Hospitalization During Specific Treatments Compared With No Antipsychotic Use in the Prevalent Cohort (Within-Individual Analysis). HR indicates Hazard Ratio with 95% confidence interval, IR incidence rate per 10 person-years. “LAI” refers to any long-acting injectable antipsychotic [LAI] (only one LAI in use), “mono” to monotherapy. “Other comp” indicates other combinations of multiple antipsychotics (two LAIs, more than two antipsychotics). “Other oral” indicates other oral antipsychotics (first generation antipsychotics, ziprasidone, asenapine). aripi: aripiprazole, clozap: clozapine, olanza: olanzapine, risper: risperidone, queti: quetiapine. Combination of two antipsychotics denoted with “—".

**Drug**	**HR (95% CI)**	**p-value**	**events**	**Person-years**	**IR (95% CI)**
no use	reference	reference	32635	188308	1.73 (1.71—1.75)
aripi—clozap	0.42 (0.39–0.46)	<0.0001	2214	7232	3.06 (2.94-3.19)
olanza— LAI	0.48 (0.44–0.51)	<0.0001	2867	7103	4.04 (3.89—4.19)
clozap mono	0.49 (0.47–0.51)	<0.0001	24319	75557	3.22 (3.18—3.26)
olanza—clozap	0.49 (0.44–0.54)	<0.0001	1370	3010	4.55 (4.32—4.80)
clozap— LAI	0.50 (0.42–0.59)	<0.0001	463	848	5.46 (4.99—5.98)
risper—clozap	0.50 (0.43–0.59)	<0.0001	1082	2359	4.59 (4.32—4.87)
clozap—queti	0.52 (0.48–0.57)	<0.0001	1912	4151	4.61 (4.40—4.82)
clozap— other oral	0.53 (0.50–0.56)	<0.0001	9489	16167	5.87 (5.75—5.99)
other oral—LAI	0.55 (0.53–0.58)	<0.0001	11436	28815	3.97 (3.90—4.04)
queti— LAI	0.55 (0.50–0.60)	<0.0001	2295	4922	4.66 (4.48—4.86)
LAI mono	0.56 (0.54–0.58)	<0.0001	13891	48976	2.84 (2.79—2.88)
other comp	0.58 (0.56–0.60)	<0.0001	29551	84201	3.51 (3.47—3.55)
olanza—queti	0.61 (0.56–0.66)	<0.0001	1696	5294	3.20 (3.05—3.36)
olanza— other oral	0.64 (0.61–0.67)	<0.0001	8176	19549	4.18 (4.09—4.27)
olanza mono	0.64 (0.61–0.66)	<0.0001	16326	64021	2.55 (2.51—2.59)
aripi—queti	0.65 (0.59–0.72)	<0.0001	845	2635	3.21 (3.00—3.43)
risper—olanza	0.65 (0.58–0.72)	<0.0001	992	3076	3.23 (3.03—3.43)
risper—queti	0.66 (0.60–0.73)	<0.0001	1455	4583	3.17 (3.02—3.34)
risper— LAI	0.67 (0.60–0.76)	<0.0001	1174	2242	5.24 (4.94—5.54)
queti— other oral	0.68 (0.63–0.74)	<0.0001	3284	9107	3.61 (3.48—3.73)
aripi— LAI	0.70 (0.59–0.83)	<0.0001	276	456	6.05 (5.38—6.81)
olanza—aripi	0.70 (0.62–0.78)	<0.0001	1000	2858	3.50 (3.29— 3.72)
other oral mono	0.71 (0.69–0.73)	<0.0001	19467	101095	1.93 (1.90—1.95)
risper— other oral	0.72 (0.68–0.76)	<0.0001	5970	15886	3.76 (3.66—3.85)
aripi— other oral	0.73 (0.60–0.88)	0.00104	648	968	6.69 (6.20—7.23)
aripi mono	0.74 (0.65–0.84)	<0.0001	1378	4576	3.01 (2.86—3.17)
risper mono	0.75 (0.72–0.79)	<0.0001	8528	35199	2.42 (2.37— 2.47)
risper—aripi	0.85 (0.62–1.16)	0.29930	96	382	2.51 (2.06—3.07)
queti mono	0.93 (0.88–0.97)	0.00283	7780	22975	3.39 (3.31—3.46)

© 2019 American Medical Association. All rights reserved.

eTable 4. The Effect Of Adding Any Other Antipsychotic On Top of Aripiprazole, Clozapine, Quetiapine, and Risperidone Monotherapy.

For example, adding another antipsychotic to aripiprazole results in about 12% lower risk of psychiatric rehospitalization (HR 0.88, 95%CI 0.72-1.06). “Effect” indicates hazard ratio, and Lo95CI and Hi95CI confidence intervals. Hospitalization outcomes were analyzed by using within-individual analysis, and mortality by using traditional Cox model. aripi: aripiprazole, clozap: clozapine, olanza: olanzapine, risper: risperidone, queti: quetiapine.

RESULT	OUTCOME	DRG	Effect	Lo95CI	Hi95CI	Pvalue
1	Psychiatric hospitalization	aripi	0.877	0.723	1.064	0.18281
2	Psychiatric hospitalization	clozap	1.003	0.929	1.082	0.9473
3	Psychiatric hospitalization	olanza	0.922	0.811	1.047	0.21042
4	Psychiatric hospitalization	queti	0.659	0.604	0.718	<1e-05
5	Psychiatric hospitalization	risper	0.874	0.785	0.973	0.01383
6	Any hospitalization	aripi	0.823	0.698	0.972	0.02166
7	Any hospitalization	clozap	1.014	0.948	1.085	0.6807
8	Any hospitalization	olanza	0.896	0.805	0.998	0.04642
9	Any hospitalization	queti	0.758	0.687	0.836	<1e-05
10	Any hospitalization	risper	0.83	0.755	0.913	0.00013
11	Death	aripi	0.601	0.421	0.858	0.00501
12	Death	clozap	0.797	0.637	0.998	0.04765
13	Death	olanza	0.81	0.743	0.882	<1e-05
14	Death	queti	0.687	0.549	0.859	0.00102
15	Death	risper	0.67	0.538	0.834	0.00035

© 2019 American Medical Association. All rights reserved.

eTable 5. Risk of Psychiatric Hospitalization During Specific Treatments Compared With No Antipsychotic Use In The Prevalent Cohort (Within-Individual Analysis). The first 90 days of all treatments are omitted to exclude switching periods. HR indicates Hazard Ratio, IR indicates incidence rate per 10 person-years. "LAI" refers to any long-acting injectable antipsychotic (only 1 used), "mono" to monotherapy, "other oral" indicates other oral antipsychotics (first generation antipsychotics, ziprasidone, asenapine), "other comp" indicates other combinations of multiple antipsychotics (two LAIs, more than two antipsychotics). aripi: aripiprazole, clozap: clozapine, olanza: olanzapine, risper: risperidone, queti: quetiapine. Combination of two antipsychotics denoted with "—".

Drug	HR (95% CI)	p-value	events	Person-years	IR (95% CI)
no use	reference	reference	59058	208221	2.84 (2.81—2.86)
aripi—clozap	0.55 (0.51–0.61)	<0.0001	2009	6748	2.98 (2.85—3.11)
olanza—LAI	0.59 (0.55–0.63)	<0.0001	2532	6623	3.82 (3.68—3.97)
olanza—clozap	0.61 (0.55–0.67)	<0.0001	1099	2742	4.01 (3.78—4.25)
risper—clozap	0.64 (0.55–0.74)	<0.0001	944	2205	4.28 (4.02—4.56)
other comp	0.65 (0.63–0.67)	<0.0001	22341	75838	2.95 (2.91—2.98)
clozap—LAI	0.65 (0.54–0.79)	<0.0001	385	781	4.93 (4.46—5.45)
olanza—queti	0.65 (0.60–0.71)	<0.0001	1302	4644	2.80 (2.66—2.96)
clozap—queti	0.67 (0.61–0.73)	<0.0001	1627	3803	4.28 (4.08—4.49)
queti—LAI	0.67 (0.61–0.74)	<0.0001	1977	4478	4.42 (4.22—4.61)
other oral—LAI	0.68 (0.65–0.70)	<0.0001	10563	27355	3.86 (3.79—3.94)
aripi—queti	0.68 (0.61–0.76)	<0.0001	648	2326	2.79 (2.58—3.01)
clozap—other oral	0.68 (0.65–0.71)	<0.0001	8519	15246	5.59 (5.47—5.71)
clozap mono	0.68 (0.66–0.70)	<0.0001	25012	75143	3.33 (3.29—3.37)
risper—aripi	0.70 (0.49–0.98)	0.04060	55	314	1.75 (1.34—2.28)
risper—olanza	0.71 (0.63–0.80)	<0.0001	758	2699	2.81 (2.62—3.02)
LAI mono	0.72 (0.69–0.74)	<0.0001	15228	49022	3.11 (3.06—3.16)
olanza—other oral	0.73 (0.70–0.77)	<0.0001	6946	18012	3.86 (3.77—3.95)
risper—queti	0.74 (0.67–0.83)	<0.0001	1193	4113	2.90 (2.74—3.07)
risper—LAI	0.75 (0.66–0.86)	<0.0001	849	1886	4.50 (4.21—4.82)
queti—other oral	0.77 (0.71–0.83)	<0.0001	2687	8179	3.29 (3.16—3.41)
olanza mono	0.80 (0.77–0.83)	<0.0001	17052	63313	2.69 (2.65—2.73)
olanza—aripi	0.81 (0.73–0.91)	0.00021	827	2527	3.27 (3.06—3.50)
risper—other oral	0.81 (0.77–0.86)	<0.0001	4956	14494	3.42 (3.33—3.52)
aripi—other oral	0.82 (0.69–0.98)	0.02814	550	846	6.50 (5.98—7.07)
aripi—LAI	0.83 (0.69–1.00)	0.04779	235	390	6.03 (5.31—6.85)
other oral mono	0.84 (0.82–0.86)	<0.0001	23516	102812	2.29 (2.26—2.32)
aripi mono	0.86 (0.78–0.94)	0.00111	1564	4573	3.42 (3.25—3.59)
risper mono	0.89 (0.85–0.92)	<0.0001	8683	34491	2.52 (2.47—2.57)
queti mono	1.03 (0.99–1.08)	0.09518	8293	22724	3.65 (3.57—3.73)

© 2019 American Medical Association. All rights reserved.

eTable 6. Risk of All-Cause Hospitalization During Specific Treatments Compared With No Antipsychotic Use in the Prevalent Cohort (Within-Individual Analysis).
HR indicates Hazard Ratio, IR indicates incidence rate per 10 person-years. “LAI” refers to any long-acting injectable antipsychotic (when only 1 used at a time), “mono” to monotherapy, “other oral” indicates other oral antipsychotics (first generation antipsychotics, ziprasidone, asenapine), “other comp” indicates other combinations of multiple antipsychotics (two LAIs, more than two antipsychotics). aripi: aripiprazole, clozap: clozapine, olanza: olanzapine, risper: risperidone, queti: quetiapine. Combination of two antipsychotics denoted with “—".

Drug	HR (95% CI)	p-value	events	Person-years	IR (95% CI)
no use	reference	reference	91452	187415	4.88 (4.85—4.91)
aripi— clozap	0.50 (0.47–0.54)	<0.0001	3498	7216	4.85 (4.69—5.01)
clozap mono	0.57 (0.55–0.59)	<0.0001	38322	75356	5.09 (5.03—5.14)
olanza—LAI	0.57 (0.54–0.60)	<0.0001	4604	7077	6.51 (6.32—6.70)
clozap—LAI	0.59 (0.50–0.68)	<0.0001	646	845	7.65 (7.08—8.26)
risper—clozap	0.59 (0.53–0.67)	<0.0001	1631	2351	6.94 (6.61—7.28)
clozap—queti	0.60 (0.56–0.65)	<0.0001	2922	4138	7.06 (6.81—7.32)
olanza—clozap	0.60 (0.55–0.65)	<0.0001	2178	2998	7.26 (6.97—7.58)
clozap—other oral	0.61 (0.58–0.64)	<0.0001	14043	16102	8.72 (8.58—8.87)
other oral—LAI	0.62 (0.59–0.64)	<0.0001	19642	28688	6.85 (6.75—6.94)
other comp	0.65 (0.63–0.66)	<0.0001	56486	83785	6.74 (6.69—6.80)
LAI mono	0.67 (0.65–0.69)	<0.0001	26602	48777	5.45 (5.39—5.52)
queti—LAI	0.67 (0.62–0.71)	<0.0001	3887	4899	7.93 (7.69—8.19)
olanza—other oral	0.73 (0.70–0.76)	<0.0001	13838	19460	7.11 (6.99—7.23)
risper—olanza	0.74 (0.68–0.81)	<0.0001	1827	3063	5.96 (5.70—6.24)
olanza—queti	0.76 (0.71–0.81)	<0.0001	3558	5269	6.75 (6.53—6.98)
risper—LAI	0.76 (0.70–0.83)	<0.0001	1971	2230	8.84 (8.46—9.24)
aripi—LAI	0.77 (0.66–0.89)	0.00047	384	455	8.45 (7.64—9.34)
olanza mono	0.77 (0.75–0.80)	<0.0001	32840	63772	5.15 (5.09—5.21)
other oral mono	0.79 (0.77–0.81)	<0.0001	56133	100514	5.58 (5.54—5.63)
olanza—aripi	0.79 (0.72–0.87)	<0.0001	1660	2849	5.83 (5.55—6.11)
aripi—other oral	0.80 (0.70–0.92)	0.00129	988	963	10.26 (9.64—10.92)
aripi—queti	0.80 (0.73–0.87)	<0.0001	1645	2624	6.27 (5.97—6.58)
queti—other oral	0.80 (0.76–0.84)	<0.0001	7076	9052	7.82 (7.64—8.00)
risper—other oral	0.80 (0.77–0.84)	<0.0001	10981	15807	6.95 (6.82—7.08)
risper—queti	0.81 (0.76–0.87)	<0.0001	3089	4559	6.78 (6.54—7.02)
risper—aripi	0.87 (0.71–1.07)	0.18491	191	380	5.02 (4.36—5.79)
aripi mono	0.90 (0.83–0.97)	0.00881	2682	4557	5.89 (5.67—6.11)
risper mono	0.91 (0.88–0.94)	<0.0001	20751	35000	5.93 (5.85—6.01)
queti mono	0.97 (0.94–1.00)	0.08048	17594	22822	7.71 (7.60—7.82)

© 2019 American Medical Association. All rights reserved.

eTable 7. Risk of Psychiatric Hospitalization During Specific Treatments Compared With No Antipsychotic Use In The Incident Cohort (Within-Individual Analysis).
HR indicates Hazard Ratio, IR indicates incidence rate per 10 person-years. "LAI" refers to any long-acting injectable antipsychotic (when only 1 used at a time), "mono" to monotherapy, "other oral" indicates other oral antipsychotics (first generation antipsychotics, ziprasidone, asenapine), "other comp" indicates other combinations of multiple antipsychotics (two LAIs, more than two antipsychotics). aripi: aripiprazole, clozap: clozapine, olanza: olanzapine, risper: risperidone, queti: quetiapine. Combination of two antipsychotics denoted with "—".

Drug	HR (95% CI)	p-value	events	Person-years	IR (95% CI)
no use	reference	reference	6592	25717	2.56 (2.50—2.63)
aripi—clozap	0.34 (0.28–0.42)	<0.0001	410	1777	2.31 (2.09—2.54)
clozap mono	0.44 (0.39–0.49)	<0.0001	2592	9489	2.73 (2.63—2.84)
olanza—queti	0.44 (0.35–0.55)	<0.0001	189	758	2.49 (2.16—2.87)
LAI mono	0.45 (0.40–0.51)	<0.0001	1127	4661	2.42 (2.28—2.56)
olanza—LAI	0.45 (0.37–0.54)	<0.0001	256	600	4.27 (3.77—4.82)
clozap—LAI	0.48 (0.22–1.04)	0.06308	60	146	4.10 (3.18—5.28)
other oral—LAI	0.49 (0.39–0.62)	<0.0001	246	621	3.96 (3.50—4.49)
olanza—clozap	0.50 (0.39–0.64)	<0.0001	142	300	4.73 (4.01—5.58)
queti—LAI	0.50 (0.37–0.67)	<0.0001	241	568	4.24 (3.74—4.81)
risper—olanza	0.51 (0.39–0.66)	<0.0001	133	368	3.61 (3.05—4.28)
other comp	0.54 (0.48–0.62)	<0.0001	1193	2100	5.68 (5.37—6.01)
olanza—other oral	0.55 (0.46–0.65)	<0.0001	411	1067	3.85 (3.50—4.24)
clozap—other oral	0.56 (0.46–0.68)	<0.0001	601	894	6.73 (6.21—7.29)
clozap—queti	0.56 (0.46–0.69)	<0.0001	331	612	5.41 (4.86—6.03)
risper—LAI	0.56 (0.38–0.83)	0.00427	51	116	4.40 (3.34—5.79)
olanza mono	0.58 (0.53–0.64)	<0.0001	2626	11565	2.27 (2.19—2.36)
aripi—LAI	0.59 (0.43–0.80)	0.00087	61	113	5.40 (4.20—6.94)
risper—aripi	0.60 (0.30–1.19)	0.14394	14	96	1.46 (0.87—2.47)
risper mono	0.60 (0.52–0.68)	<0.0001	1052	5548	1.90 (1.79—2.01)
risper—other oral	0.62 (0.52–0.76)	<0.0001	310	688	4.51 (4.03—5.04)
risper—clozap	0.65 (0.33–1.28)	0.21303	102	153	6.66 (5.49—8.09)
queti—other oral	0.67 (0.57–0.79)	<0.0001	356	773	4.61 (4.15—5.11)
aripi—queti	0.68 (0.53–0.88)	0.00330	174	651	2.67 (2.30—3.10)
other oral mono	0.70 (0.63–0.78)	<0.0001	1127	4216	2.67 (2.52-2.83)
olanza—aripi	0.70 (0.55–0.91)	0.00737	211	684	3.08 (2.70—3.53)
aripi mono	0.75 (0.50–1.12)	0.16285	353	1510	2.34 (2.11—2.60)
queti mono	0.76 (0.67–0.86)	<0.0001	1218	4298	2.83 (2.68—3.00)
risper—queti	0.80 (0.56–1.14)	0.22146	170	590	2.88 (2.48—3.35)
aripi—other oral	1.04 (0.75–1.45)	0.80920	72	135	5.33 (4.23—6.72)

© 2019 American Medical Association. All rights reserved.

eTable 8. Risk of Somatic Hospitalization During Specific Treatments Compared With No Antipsychotic Use (Within-Individual Analysis).

HR indicates Hazard Ratio, IR incidence rate per 10 person-years. "LAI" refers to any long-acting injectable antipsychotic (when only 1 used at a time), "mono" to monotherapy, "other oral" indicates other oral antipsychotics (first generation antipsychotics, ziprasidone, asenapine), "other comp" indicates other combinations of multiple antipsychotics (two LAIs, more than two antipsychotics). aripi: aripiprazole, clozap: clozapine, olanza: olanzapine, risper: risperidone, queti: quetiapine. Combination of two antipsychotics denoted with "—".

Drug	HR (95% CI)	p-value	events	Person-years	IR (95% CI)
no use	reference	reference	49717	188107	2.64 (2.62–2.67)
other oral–LAI	0.79 (0.75–0.84)	<0.0001	7615	28912	2.63 (2.58–2.69)
other comp	0.82 (0.79–0.85)	<0.0001	24360	84390	2.89 (2.85–2.92)
LAI mono	0.92 (0.88–0.97)	0.00127	11824	49047	2.41 (2.37–2.45)
risper–other oral	0.92 (0.87–0.98)	0.00962	4385	15935	2.75 (2.67–2.83)
olanza–LAI	0.93 (0.85–1.01)	0.09244	1656	7134	2.32 (2.21–2.44)
other oral mono	0.94 (0.91–0.97)	0.00017	31883	100984	3.16 (3.12–3.19)
clozap–other oral	0.95 (0.89–1.02)	0.17421	4309	16273	2.65 (2.57–2.73)
olanza–other oral	0.95 (0.90–1.01)	0.10180	5083	19629	2.59 (2.52–2.66)
risper–LAI	0.97 (0.86–1.09)	0.58966	767	2253	3.40 (3.17–3.65)
risper–olanza	0.98 (0.86–1.12)	0.77643	786	3082	2.55 (2.38–2.74)
queti–other oral	0.99 (0.93–1.06)	0.78163	3092	9127	3.39 (3.27–3.51)
aripi–LAI	1.00 (0.76–1.33)	0.97282	100	460	2.17 (1.79–2.64)
queti–LAI	1.02 (0.93–1.12)	0.65384	1370	4942	2.77 (2.63–2.92)
clozap mono	1.04 (0.99–1.10)	0.12022	13705	75793	1.81 (1.78–1.84)
aripi–other oral	1.05 (0.88–1.25)	0.61762	293	977	3.00 (2.68–3.36)
aripi–clozap	1.06 (0.95–1.19)	0.28035	1283	7259	1.77 (1.67–1.87)
olanza mono	1.09 (1.04–1.14)	0.00019	14400	64113	2.25 (2.21–2.28)
queti mono	1.09 (1.04–1.15)	0.00055	7702	23004	3.35 (3.27–3.42)
olanza queti	1.10 (1.00–1.21)	0.06209	1477	5308	2.78 (2.64–2.93)
risper mono	1.11 (1.06–1.17)	<0.0001	10836	35191	3.08 (3.02–3.14)
risper–queti	1.11 (1.01–1.22)	0.02372	1435	4591	3.13 (2.97–3.29)
clozap–queti	1.12 (0.99–1.26)	0.06898	955	4177	2.29 (2.15–2.44)
olanza–clozap	1.12 (0.98–1.29)	0.10387	819	3023	2.71 (2.53–2.90)
risper–clozap	1.12 (0.96–1.32)	0.15978	547	2369	2.31 (2.12–2.51)
olanza–aripi	1.15 (0.98–1.34)	0.08460	556	2869	1.94(1.78–2.11)
aripi–queti	1.17 (1.03–1.34)	0.02005	645	2643	2.44 (2.26–2.64)
clozap–LAI	1.17 (0.89–1.53)	0.26010	173	853	2.03 (1.75–2.35)
aripi mono	1.18 (1.04–1.33)	0.00889	1017	4587	2.22 (2.09–2.36)
risper–aripi	1.26 (0.91–1.75)	0.16040	76	383	1.99 (1.59–2.49)

© 2019 American Medical Association. All rights reserved.

eTable 9. Risk Of Death During Specific Antipsychotic Treatments Compared With No Use of Antipsychotic in the Total Cohort (Between-Individual Analysis). Hospital periods with over 7 days duration were censored. HR indicates Hazard Ratio, IR incidence rate per 10,000 person-years. "LAI" refers to any long-acting injectable antipsychotic (when only 1 used at a time), "mono" to monotherapy, "other oral" indicates other oral antipsychotics (first generation antipsychotics, ziprasidone, asenapine), "other comp" indicates other combinations of multiple antipsychotics (two LAIs, more than two antipsychotics). aripi: aripiprazole, clozap: clozapine, olanza: olanzapine, risper: risperidone, queti: quetiapine. Combination of two antipsychotics denoted with "—".

Drug	HR (95% CI)	p-value	events	Person-years	IR (95% CI)
no use	reference	reference	5635	187774	300 (292—308)
aripi—queti	0.15 (0.08–0.28)	<0.0001	10	2632	38 (21—71)
clozap—queti	0.22 (0.15–0.34)	<0.0001	22	4153	53 (35—81)
aripi—LAI	0.23 (0.07–0.73)	0.01283	3	457	66 (21—204)
olanza—clozap	0.25 (0.17–0.39)	<0.0001	22	3010	73 (48—111)
aripi—clozap	0.27 (0.20–0.38)	<0.0001	37	7236	51 (37—71)
olanza—aripi	0.26 (0.16–0.41)	<0.0001	19	2856	67 (42—104)
risper—clozap	0.26 (0.16–0.42)	<0.0001	18	2360	76 (48—121)
risper—queti	0.27 (0.21–0.36)	<0.0001	55	4574	120 (92—156)
risper—aripi	0.29 (0.10–0.89)	0.03002	3	381	79 (25—244)
olanza—LAI	0.33 (0.26–0.41)	<0.0001	89	7102	125 (102—154)
olanza—other oral	0.35 (0.31–0.40)	<0.0001	247	19536	126 (112—143)
queti—other oral	0.36 (0.30–0.43)	<0.0001	129	9087	142 (120—169)
clozap mono	0.39 (0.35–0.43)	<0.0001	600	75576	79 (73—86)
risper—olanza	0.41 (0.31–0.56)	<0.0001	46	3072	150 (112—200)
olanza mono	0.41 (0.38–0.45)	<0.0001	808	63934	126 (118—135)
olanza—queti	0.41 (0.32–0.52)	<0.0001	76	5284	144 (115—180)
other comp	0.42 (0.39–0.45)	<0.0001	1288	84087	153 (145—162)
clozap—other oral	0.42 (0.36–0.49)	<0.0001	176	16191	109 (94—126)
aripi mono	0.43(0.31–0.58)	<0.0001	42	4569	92 (68—124)
other oral—LAI	0.44 (0.39–0.49)	<0.0001	464	28796	161 (147—176)
risper—other oral	0.44 (0.38–0.50)	<0.0001	251	15863	158 (140—179)
queti—LAI	0.44 (0.36–0.56)	<0.0001	87	4920	177 (143—218)
queti mono	0.43 (0.39–0.49)	<0.0001	385	22897	168 (152—186)
clozap—LAI	0.48 (0.27–0.84)	0.01004	11	848	130 (72—234)
LAI mono	0.53 (0.49–0.58)	<0.0001	865	48908	177 (166— 189)
risper mono	0.51 (0.46–0.55)	<0.0001	655	35101	187 (173—201)
other oral mono	0.55 (0.51–0.58)	<0.0001	1786	100778	177 (169—186)
risper—LAI	0.52 (0.39–0.71)	<0.0001	52	2240	232 (177— 305)
aripi—other oral	0.62 (0.39–1.00)	0.05007	18	969	186 (117—295)

© 2019 American Medical Association. All rights reserved.

eTable 10. Risk of Psychiatric Hospitalization During Specific Antipsychotic Treatments Compared With No Use of Antipsychotic in the Total Cohort (Between-Individual Analysis).
HR indicates Hazard Ratio, IR incidence rate per 10 person-years. "LAI" refers to any long-acting injectable antipsychotic (when only 1 used at a time), "mono" to monotherapy, "other oral" indicates other oral antipsychotics (first generation antipsychotics, ziprasidone, asenapine), "other comp" indicates other combinations of multiple antipsychotics (two LAIs, more than two antipsychotics). aripi: aripiprazole, clozap: clozapine, olanza: olanzapine, risper: risperidone, queti: quetiapine. Combination of two antipsychotics denoted with "—".

**Drug**	**HR (95% CI)**	**p-value**	**events**	**Person-years**	**IR (95% CI)**
no use	reference	reference	32635	188308	1.73 (1.71—1.75)
olanza—LAI	0.57 (0.51–0.63)	<0.0001	2867	7103	4.04 (3.89—4.19)
clozap mono	0.60 (0.57–0.63)	<0.0001	24319	75557	3.22 (3.18—3.26)
aripi—clozap	0.61 (0.55–0.68)	<0.0001	2214	7232	3.06 (2.94—3.19)
olanza mono	0.62 (0.59–0.65)	<0.0001	16326	64021	2.55 (2.51—2.59)
olanza—clozap	0.65 (0.58–0.73)	<0.0001	1370	3010	4.55 (4.32—4.80)
LAI mono	0.66 (0.63–0.70)	<0.0001	13891	48976	2.84 (2.79—2.88)
olanza—queti	0.67 (0.61–0.75)	<0.0001	1696	5294	3.20 (3.05—3.36)
other oral—LAI	0.68 (0.64–0.72)	<0.0001	11436	28815	3.97 (3.90—4.04)
clozap—LAI	0.68 (0.53–0.87)	0.00221	463	848	5.46 (4.99—5.98)
olanza—other oral	0.68 (0.63–0.73)	<0.0001	8176	19549	4.18 (4.09—4.27)
risper—clozap	0.68 (0.55–0.84)	0.00025	1082	2359	4.59 (4.32—4.87)
risper—olanza	0.70 (0.60–0.81)	<0.0001	992	3076	3.23 (3.03—3.43)
clozap—queti	0.72 (0.64–0.81)	<0.0001	1912	4152	4.61 (4.40—4.82)
risper mono	0.74 (0.69–0.78)	<0.0001	8528	35199	2.42 (2.37—2.47)
other oral mono	0.76 (0.73–0.79)	<0.0001	19467	101095	1.93 (1.90—1.95)
other comp	0.76 (0.73–0.80)	<0.0001	29551	84201	3.51 (3.47—3.55)
aripi—queti	0.76 (0.68–0.86)	<0.0001	845	2635	3.21 (3.00—3.43)
clozap—other oral	0.76 (0.70–0.82)	<0.0001	9489	16167	5.87 (5.75—5.99)
risper—aripi	0.76 (0.60–0.98)	0.03408	96	382	2.51 (2.06—3.07)
risper—other oral	0.78 (0.73–0.84)	<0.0001	5970	15886	3.76 (3.66—3.85)
risper—queti	0.80 (0.70–0.92)	0.00150	1455	4583	3.17 (3.02—3.34)
queti—LAI	0.83 (0.74–0.93)	0.00178	2295	4922	4.66 (4.48—4.86)
aripi mono	0.84 (0.74–0.95)	0.00663	1378	4576	3.01 (2.86—3.17)
risper—LAI	0.85 (0.75–0.96)	0.01000	1174	2242	5.24 (4.94—5.54)
olanza—aripi	0.88 (0.76–1.01)	0.07596	1000	2858	3.50 (3.29—3.70)
queti—other oral	0.89 (0.82–0.96)	0.00460	3284	9107	3.61 (3.48—3.73)
aripi—LAI	0.98 (0.78–1.23)	0.83226	276	456	6.05 (5.38—6.81)
queti mono	0.99 (0.93–1.05)	0.71385	7780	22975	3.39 (3.31—3.46)
aripi—other oral	1.32 (1.06–1.65)	0.01377	648	968	6.69 (6.20—7.23)

eTable 11. Risk of Psychiatric Re-Hospitalization During Specific Treatments Compared With No Antipsychotic Treatment in the Incident Cohort (Between-Individual Analyses).
HR indicates Hazard Ratio, IR incidence rate per 10 person-years. "LAI" refers to any long-acting injectable antipsychotic (when only 1 used at a time), "mono" to monotherapy, "other oral" indicates other oral antipsychotics (first generation antipsychotics, ziprasidone, asenapine), "other comp" indicates other combinations of multiple antipsychotics (two LAIs, more than two antipsychotics). aripi: aripiprazole, clozap: clozapine, olanza: olanzapine, risper: risperidone, queti: quetiapine. Combination of two antipsychotics denoted with "—".

**Drug**	**HR (95% CI)**	**p-value**	**events**	**Person-years**	**IR (95% CI)**
no use	reference	reference	6592	25717	2.56 (2.50—2.63)
LAI mono	0.53 (0.45–0.61)	<0.0001	1127	4661	2.42 (2.28—2.56)
aripi—clozap	0.53 (0.42–0.66)	<0.0001	410	1777	2.31 (2.09—2.54)
other oral—LAI	0.59 (0.46–0.76)	<0.0001	246	621	3.96 (3.50—4.49)
clozap mono	0.59 (0.52–0.68)	<0.0001	2592	9489	2.73 (2.63—2.84)
olanza—LAI	0.59 (0.47–0.74)	<0.0001	256	600	4.27 (3.77—4.82)
olanza—queti	0.59 (0.45–0.77)	<0.0001	189	758	2.49 (2.16—2.87)
risper—aripi	0.59 (0.32–1.07)	0.08114	14	96	1.46 (0.87—2.47)
risper mono	0.63 (0.56–0.70)	<0.0001	1052	5548	1.90 (1.79—2.01)
olanza mono	0.66 (0.59–0.74)	<0.0001	2626	11565	2.27 (2.19—2.36)
risper—olanza	0.66 (0.47–0.94)	0.01922	133	368	3.61 (3.05—4.28)
olanza—other oral	0.70 (0.59–0.83)	<0.0001	411	1067	3.85 (3.50—4.24)
queti—LAI	0.69 (0.47–1.02)	0.06045	241	568	4.24 (3.74—4.81)
clozap—LAI	0.75 (0.40–1.41)	0.36887	60	146	4.10 (3.18—5.28)
aripi mono	0.78 (0.59–1.02)	0.07323	353	1510	2.34 (2.11—2-60)
risper—LAI	0.80 (0.56–1.15)	0.23610	51	116	4.40 (3.34—5.79)
other oral mono	0.81 (0.72–0.91)	0.00035	1127	4216	2.67 (2.52—2.83)
olanza—clozap	0.81 (0.58–1.13)	0.21583	142	300	4.73 (4.01—5.58)
aripi—queti	0.84 (0.64–1.09)	0.18882	174	651	2.67 (2.30—3.10)
queti mono	0.87 (0.76–0.98)	0.02784	1218	4298	2.83 (2.68—3.00)
other comp	0.91 (0.78–1.06)	0.21512	1193	2100	5.68 (5.37—6.01)
clozap—queti	0.91 (0.72–1.16)	0.46204	331	612	5.41 (4.86—6.03)
aripi—LAI	0.92 (0.59–1.44)	0.72390	61	113	5.40 (4.20—6.94)
risper—other oral	0.93 (0.63–1.36)	0.69604	310	687	4.51 (4.03—5.04)
olanza—aripi	0.96 (0.64–1.44)	0.83466	211	684	3.08 (2.70—3.53)
risper—queti	0.96 (0.67–1.37)	0.82945	170	590	2.88 (2.48—3.35)
clozap—other oral	1.05 (0.84–1.31)	0.69427	601	894	6.73 (6.21—7.29)
queti—other oral	1.05 (0.82–1.34)	0.69244	356	773	4.61 (4.15—5.11)
risper—clozap	1.17 (0.58–2.38)	0.66015	102	153	6.66 (5.49—8.09)
aripi—other oral	1.23 (0.86–1.78)	0.26079	72	135	5.33 (4.23—6.72)

© 2019 American Medical Association. All rights reserved.

eFigure 1. Restructuring of Data for Within Individual Analyses (with time resetting after each outcome event).

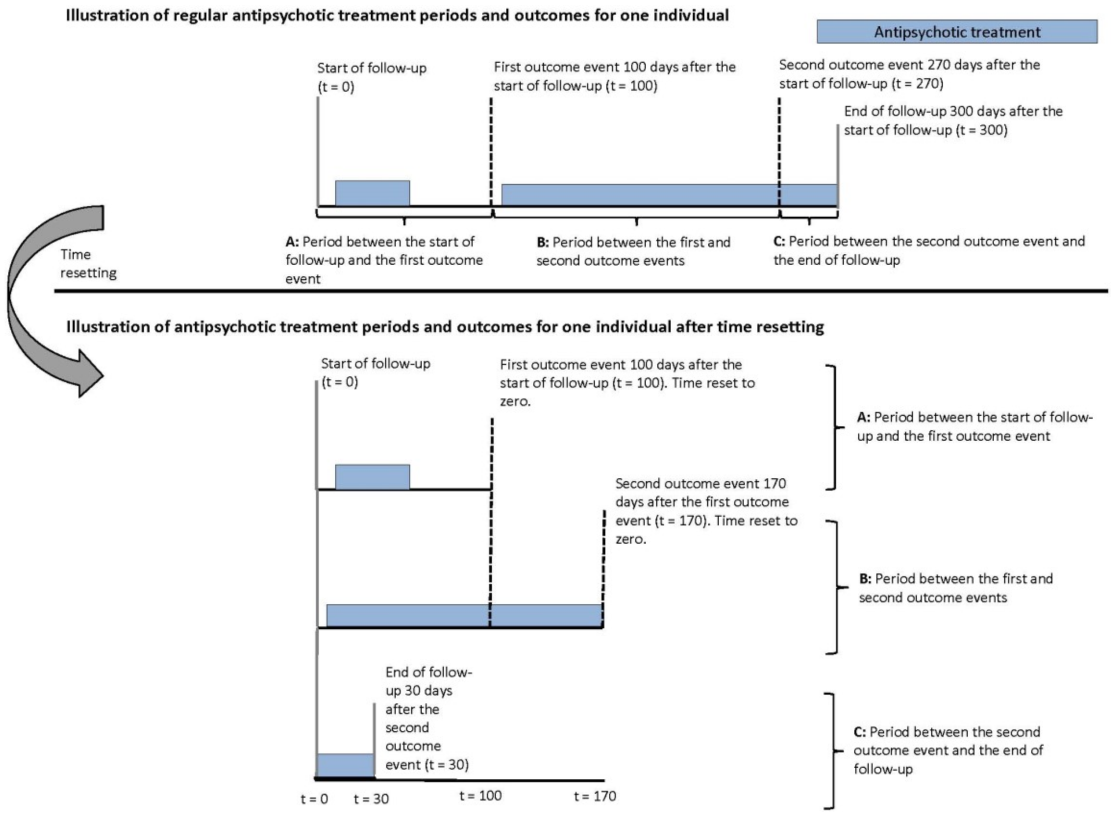

Illustration of data-analysis after time resetting by using each individual as his/her own control (continued)

In the within-individual analyses stratified Cox model is used. Each individual forms his/her own stratum. Within each stratum, time periods from the same individual resulting after time resetting are used in comparisons as different individuals are compared in a traditional Cox model. The figure below illustrates how the likelihood contribution of one individual is calculated in the within-individual model. The full likelihood is a product over all individual contributions. The method is also illustrated in Supplement to: Lichtenstein P, Halldner L, Zetterqvist J, et al. Medication for attention deficit–hyperactivity disorder and criminality. N Engl J Med 2012;367:2006-14. DOI: 10.1056/NEJMoa1203241.

In this illustration it is assumed that at any given time t an individual may have either of two possible statuses (0 or 1), denoted X(t). The status is X(t) = 0 when the individual is not treated with any antipsychotic and X(t) = 1 when treated. The individual is also assumed to have his/her own baseline hazard that is proportional to exp(X(t)*b). In general, at the time of an event, the risk set consists of all ongoing sub-periods, i.e., of those that (after time resetting) began before the event and ended after it. The illustrated individual has two events from which his/her likelihood contribution arises. At the time of the first event (t = 100), the risk set consists also of (sub)period **B.** At the time of the second event (t = 170) there are no other ongoing sub-periods. Individual contributions of each of the two events are given in the figure and yield a total contribution of [1/(1 + exp(b))] x 1.

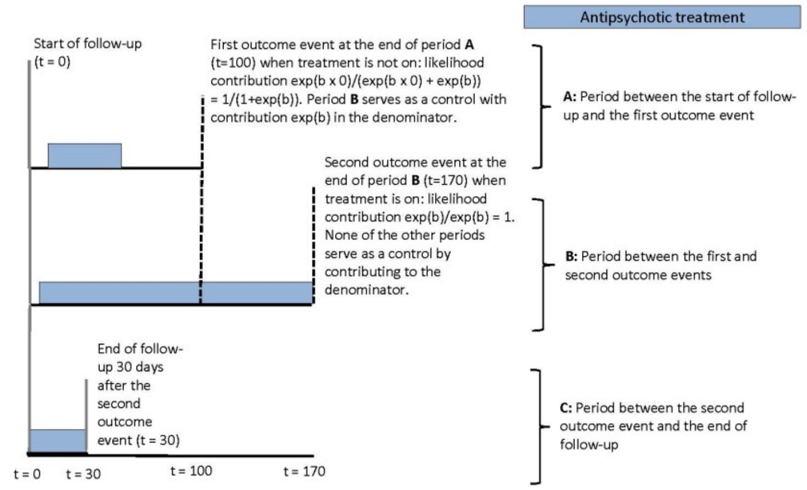

© 2019 American Medical Association. All rights reserved.

eFigure 2. Risk of Psychiatric Hospitalization During Specific Treatments Compared With No Antipsychotic Use in the Prevalent Cohort (Within-Individual Analysis). The first 90 days of all treatments are omitted to exclude switching periods. LAI indicates long-acting injection. Monotherapies are highlighted in red.

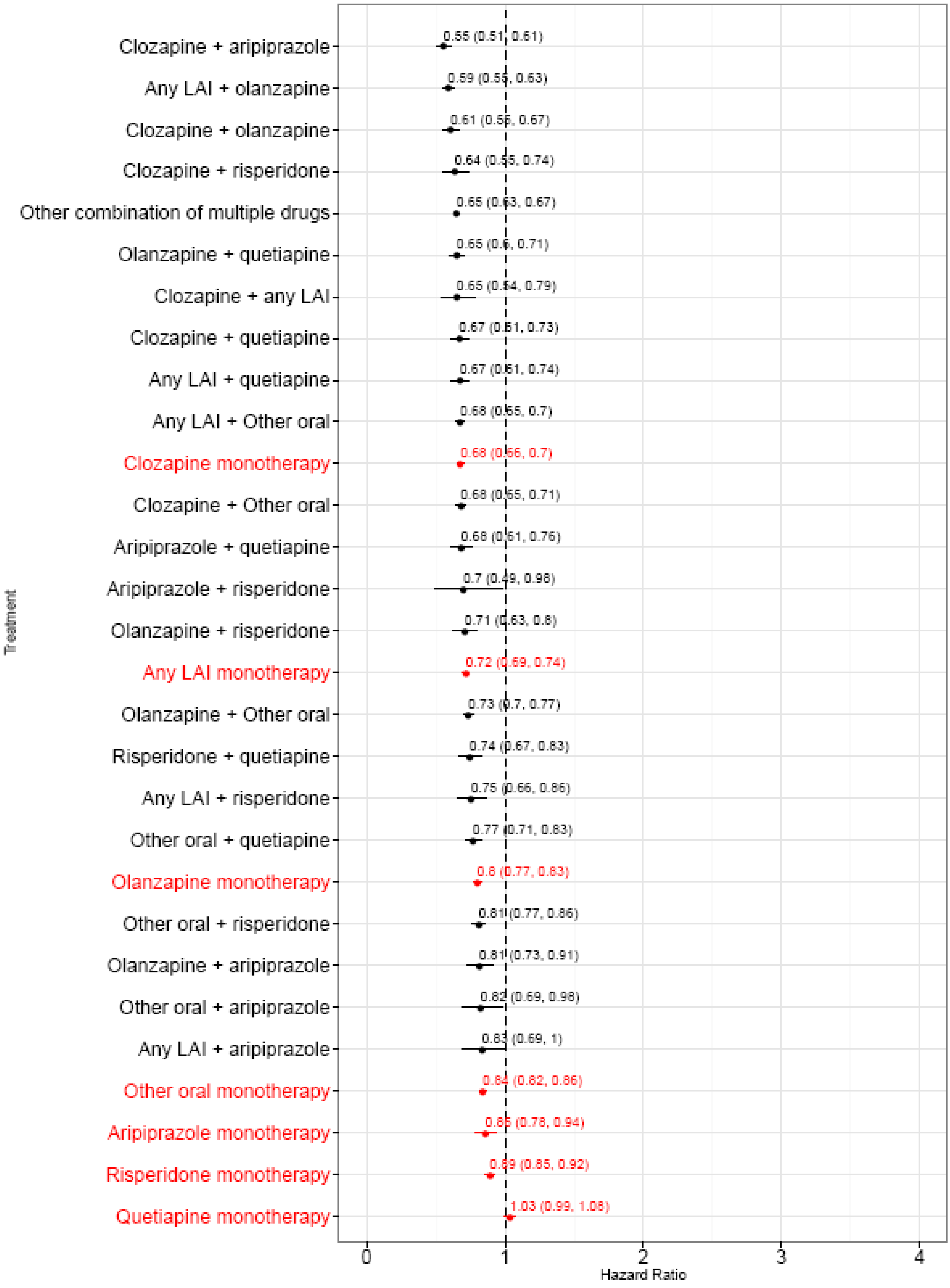

© 2019 American Medical Association. All rights reserved.

eFigure 3. Risk of All-Cause Hospitalization During Specific Treatments Compared With No Antipsychotic Use in the Prevalent Cohort (Within-Individual Analysis). LAI indicates long-acting injection. Monotherapies are highlighted in red.

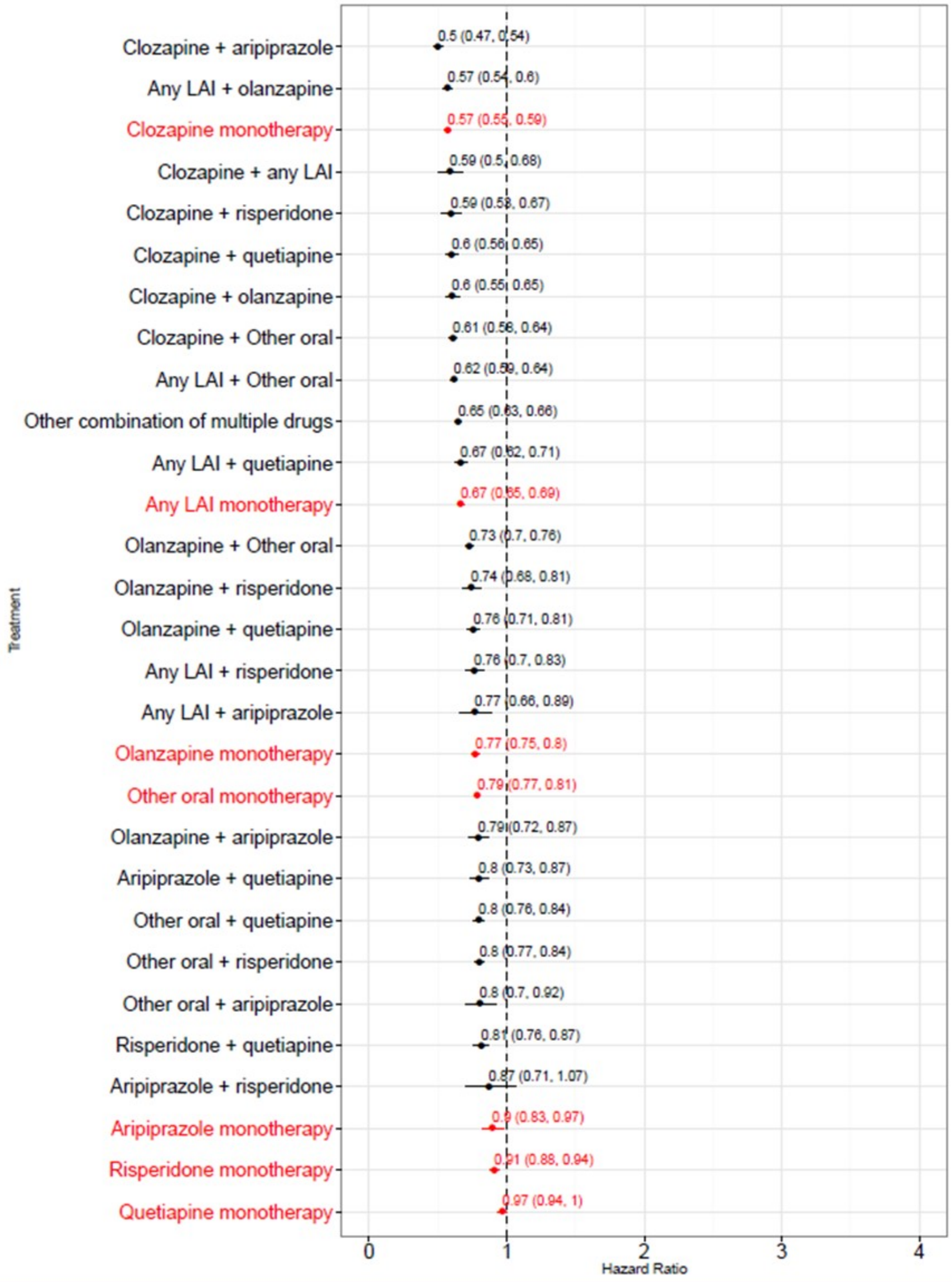

© 2019 American Medical Association. All rights reserved.

eFigure 4. Risk of Somatic Hospitalization During Specific Treatments Compared With No Antipsychotic Use (Within-Individual Analysis). Monotherapies are highlighted in red.

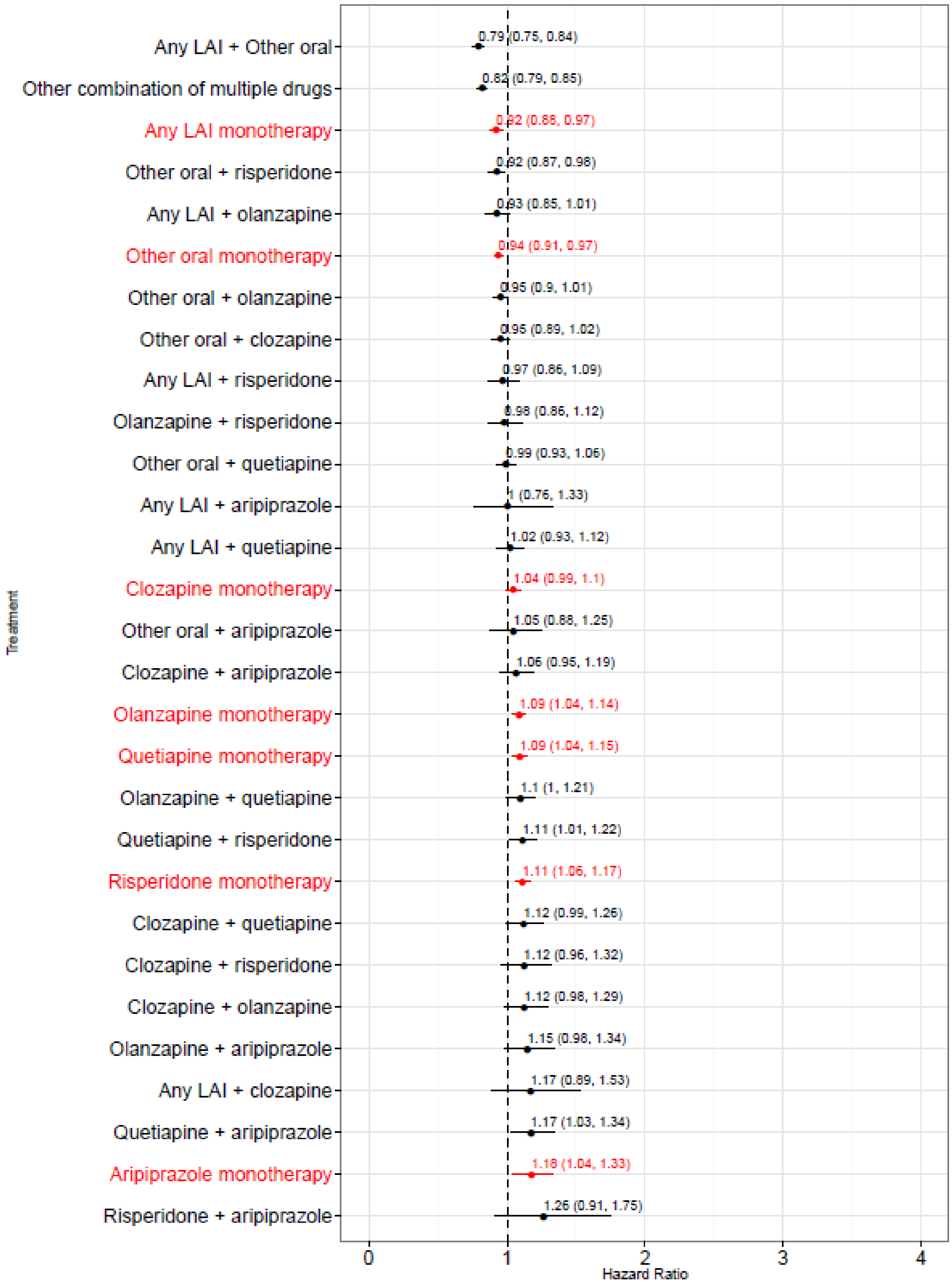

© 2019 American Medical Association. All rights reserved.

eFigure 5. Risk of Death During Specific Antipsychotic Treatments Compared With No Use of Antipsychotic in the Total Cohort (Between-Individual Analysis). Hospital periods with over 7 days duration were censored. Monotherapies are highlighted in red.

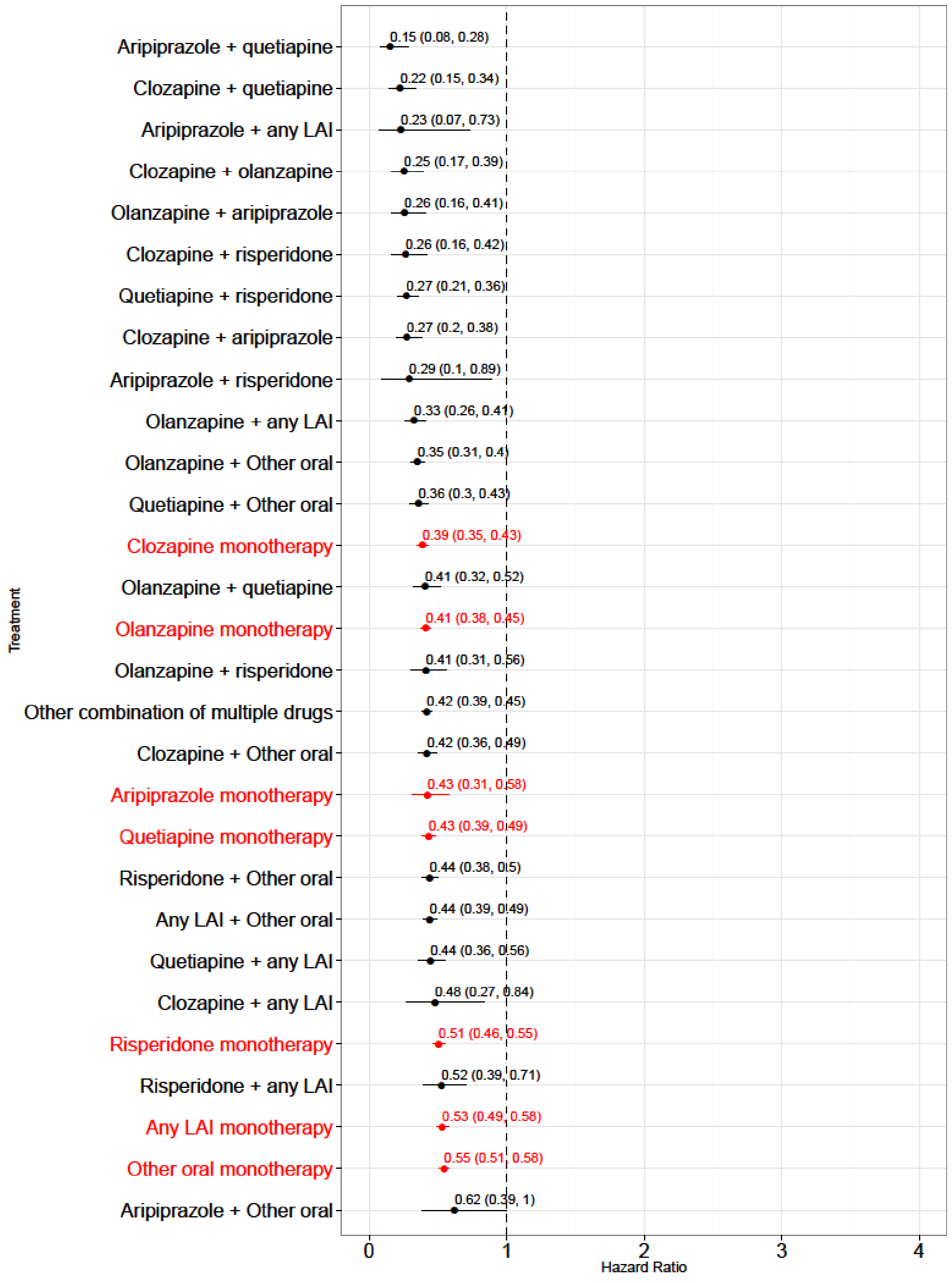

© 2019 American Medical Association. All rights reserved.

eFigure 6. Risk of Psychiatric Hospitalization During Specific Antipsychotic Treatments Compared With No Use of Antipsychotic in the Total Cohort (Between-Individual Analysis). Monotherapies are highlighted in red.

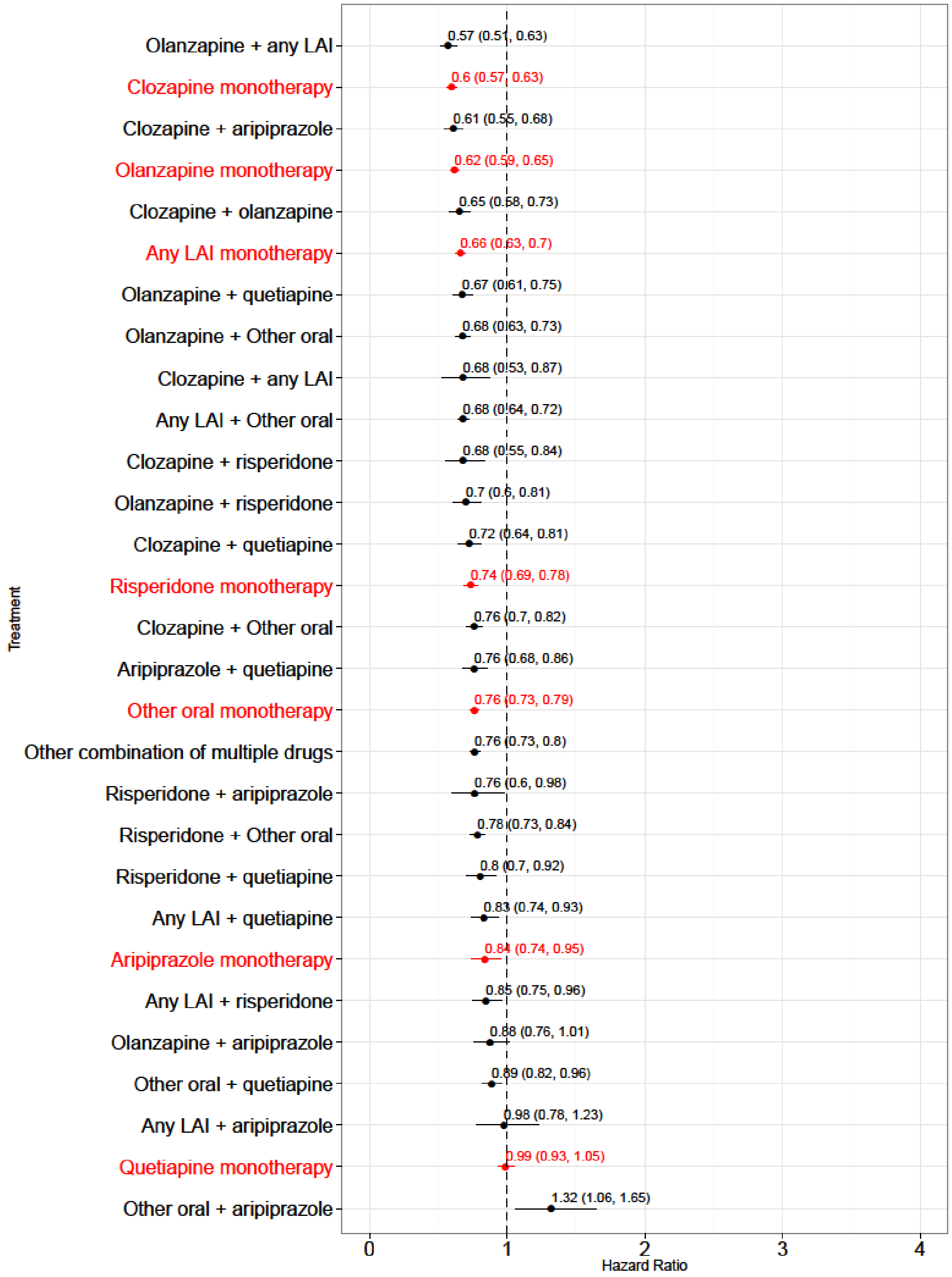

© 2019 American Medical Association. All rights reserved.

eFigure 7. Risk of Psychiatric Re-Hospitalization During Specific Treatments Compared With No Antipsychotic Treatment in the Incident Cohort (Between-Individual Analyses). Monotherapies are highlighted in red.

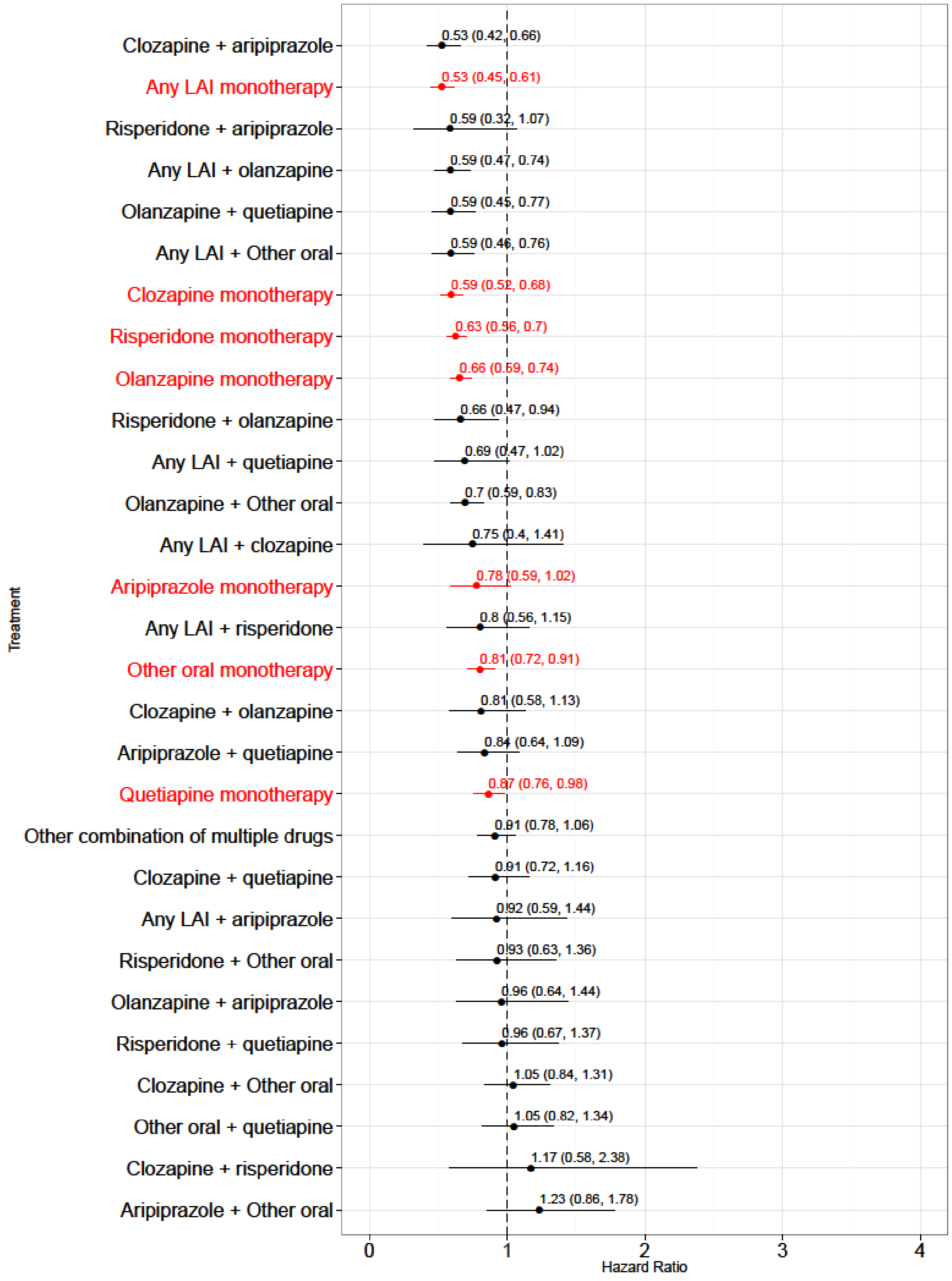

© 2019 American Medical Association. All rights reserved.

eReferences

1. Taipale H, Mehtälä J, Tanskanen A, Tiihonen J. Comparative effectiveness of antipsychotic drugs for rehospitalization in schizophrenia - a nationwide study with 20-year follow-up. *Schizophr Bull*. 2018;44(6):1381-1387 doi: 10.1093/schbul/sbx176.
2. Tiihonen J, Tanskanen A, Taipale H. 20-year nationwide follow-up study on discontinuation of antipsychotic treatment in first-episode schizophrenia. *Am J Psychiatry*. 2018;175(8):765-773.doi: 10.1176/appi.ajp.2018.17091001.

© 2019 American Medical Association. All rights reserved.
